# Aryl hydrocarbon receptor sulfenylation promotes glycogenolysis and rescues cancer chemoresistance

**DOI:** 10.1172/JCI170753

**Published:** 2023-12-15

**Authors:** Nannan Zhou, Jie Chen, Zheng Ling, Chaoqi Zhang, Yabo Zhou, Dianheng Wang, Li Zhou, Zhenfeng Wang, Nan Sun, Xin Wang, Huafeng Zhang, Ke Tang, Jingwei Ma, Jiadi Lv, Bo Huang

**Affiliations:** 1 Department of Immunology and National Key Laboratory of Medical Molecular Biology, Institute of Basic Medical Sciences;; 2 Department of Thoracic Surgery, National Cancer Center/Cancer Hospital; and; 3 Department of Breast Surgical Oncology, National Cancer Center/Cancer Hospital, Chinese Academy of Medical Sciences (CAMS) & Peking Union Medical College, Beijing, China.; 4 Department of Pathology,; 5 Department of Biochemistry and Molecular Biology, and; 6 Department of Immunology, Tongji Medical College, Huazhong University of Science and Technology, Wuhan, China.

**Keywords:** Cell Biology, Metabolism, Cancer, Cell stress, Toxins/drugs/xenobiotics

## Abstract

Elevation of reactive oxygen species (ROS) levels is a general consequence of tumor cells’ response to treatment and may cause tumor cell death. Mechanisms by which tumor cells clear fatal ROS, thereby rescuing redox balance and entering a chemoresistant state, remain unclear. Here, we show that cysteine sulfenylation by ROS confers on aryl hydrocarbon receptor (AHR) the ability to dissociate from the heat shock protein 90 complex but to bind to the PPP1R3 family member PPP1R3C of the glycogen complex in drug-treated tumor cells, thus activating glycogen phosphorylase to initiate glycogenolysis and the subsequent pentose phosphate pathway, leading to NADPH production for ROS clearance and chemoresistance formation. We found that basic ROS levels were higher in chemoresistant cells than in chemosensitive cells, guaranteeing the rapid induction of AHR sulfenylation for the clearance of excess ROS. These findings reveal that AHR can act as an ROS sensor to mediate chemoresistance, thus providing a potential strategy to reverse chemoresistance in patients with cancer.

## Graphical abstract

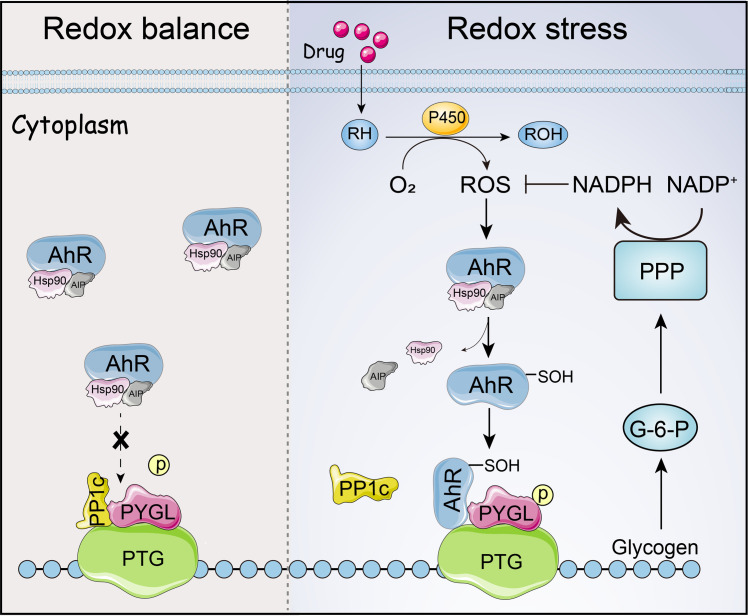

## Introduction

Cells burn sugars and fatty acids to harvest free energy for living, which, however, inevitably results in the production of reactive oxygen species (ROS), the harmful derivatives of molecular oxygen that damage lipids, proteins, and DNA and may cause cell death (1–3). Therefore, cells have to evolve a complete set of machinery to dispose of ROS and maintain their longevity. The pentose phosphate pathway (PPP) is fundamental for ROS clearance, because PPP-yielded NADPH donates hydrogen atoms, which leads to the conversion of H_2_O_2_ into H_2_O through the glutathione and thioredoxin systems (1, 4). Despite the importance of the PPP in ROS clearance, recent studies showed that glycogen-derived glucose-6-phosphatase (G6P) initiates the PPP, as evidenced in memory T cells, macrophages, and tumor cells (5–7). Thus, glycogen degradation (glycogenolysis), conventionally thought to provide glucose for glycolysis, may have an antioxidative role via the PPP. Glycogenolysis is a means of energy supply, which is regulated by hormones such as glucagon or epinephrine (8, 9). However, in vitro–cultured tumor cells or immune cells can continue glycogenolysis in the absence of hormones, suggesting that an unknown mechanism that regulates glycogenolysis is most likely involved in ROS clearance.

Aryl hydrocarbon receptor (AHR) was initially identified to sense toxic agents in 1976 (10–12). As a critical cytosolic transcription factor, AHR acts as an exposome receptor that maintains cellular homeostasis via detoxification by transactivating cytochrome P450s (CYP1A1, CYP1A2, and CYP1B1) in various cell types (13–16). As heme-containing monooxygenases, P450s catalyze the incorporation of 1 oxygen atom from molecular oxygen into the toxic molecule RH, yielding ROH and the by-product ROS (17–19), thus generating a contradictory consequence (xenobiotic detoxification versus toxic ROS generation). To reconcile this contradiction, we assume that AHR not only transactivates P450 for xenobiotic detoxification but also contributes to P450-derived ROS clearance by regulating the glycogenolysis/PPP pathway for cell survival. This assumption can be supported by chemo drug killing of tumor cells, which commonly increases AHR expression. Once entering, chemo molecules activate the P450 system in tumor cells. If the detoxification efficiency is low, tumor cells die from the direct cytotoxicity of chemo drugs; however, the high P450 efficiency results in abundant ROS production, which may also cause cell death owing to a weak clearance of ROS. Therefore, only cells with both high P450 activity and the high ability to clear ROS can survive and exhibit chemoresistance, a fatal problem in clinical cancer treatments. In this study, we provide evidence that in response to increased ROS, AHR is sulfenylated and thus binds to PPP1R3 family member PPP1R3C (PTG), thus promoting glycogen phosphorylase activity by inhibiting the protein phosphatase 1 catalytic subunit (PP1c), leading to active glycogenolysis and the shunt of G6P to the PPP.

## Results

### Drug-resistant tumor cells use higher levels of basic ROS to produce NADPH during treatment.

**Figure 1:**
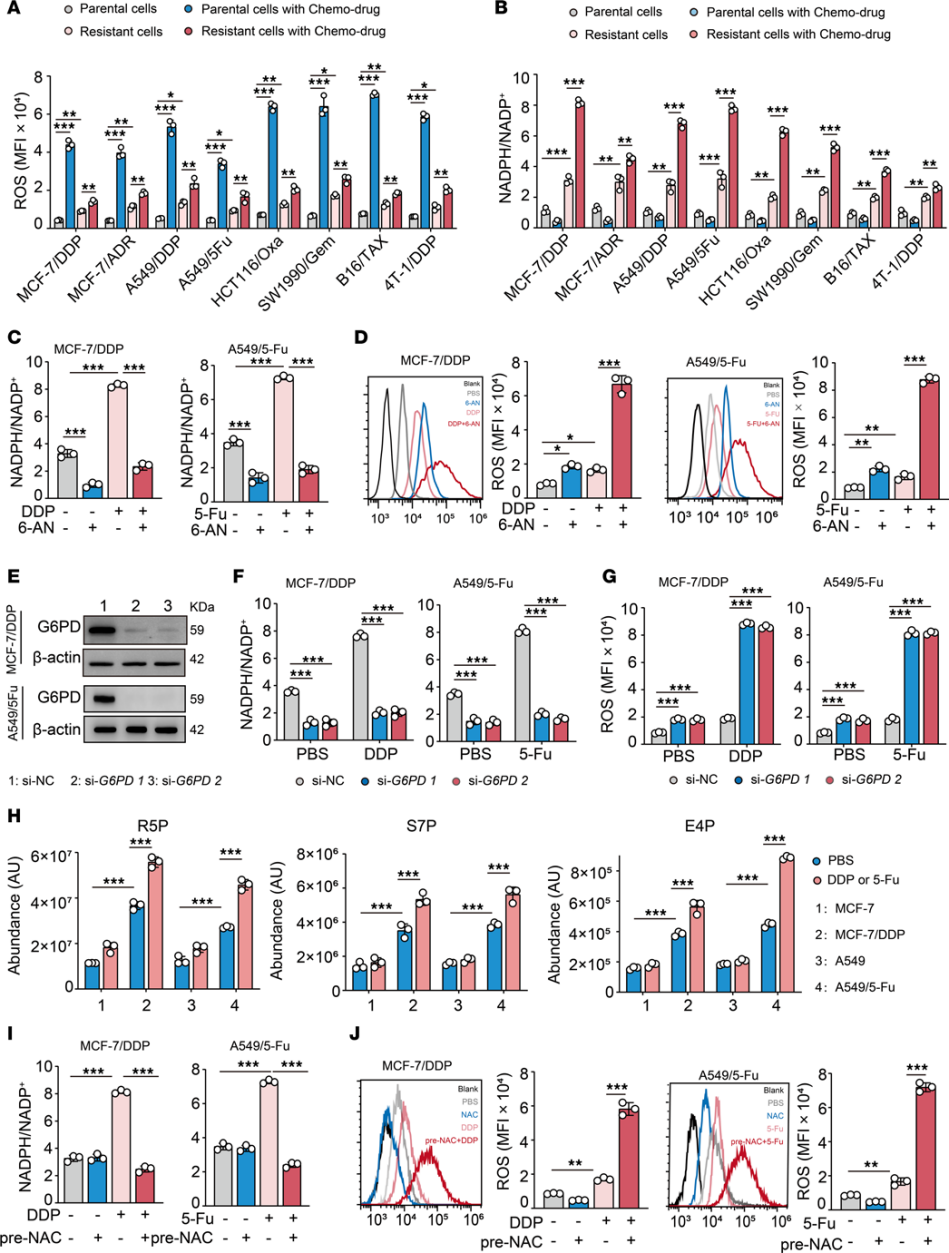
Drug-resistant tumor cells use higher ROS to produce NADPH during treatment. (**A** and **B**) Different drug-resistant tumor cell lines including MCF-7/DDP, MCF-7/ADR, A549/DDP, A549/5-Fu, HCT116/Oxa, SW1990/Gem, B16/TAX, and 4T-1/DDP and their parental tumor cells were treated with their corresponding chemo drugs for 24 hours. ROS levels (**A**) and the ratio of NADPH/NADP^+^ (**B**) were analyzed. (**C** and **D**) MCF-7/DDP and A549/5-Fu, respectively, were treated with DDP (20 μM) or 5-Fu (100 μM) alone or in combination with 6-AN (50 μM) for 24 hours. NADPH/NADP^+^ (**C**) and ROS levels (**D**) were analyzed. (**E**) The expression of G6PD in MCF-7/DDP and A549/5-Fu cells transduced with si-NC or si-*G6PD* was analyzed by Western blot. (**F** and **G**) MCF-7/DDP and A549/5-Fu cells transduced with si-NC or si-*G6PD* were treated with DDP or 5-Fu for 24 hours. NADPH/NADP^+^ (**F**) and ROS levels (**G**) were analyzed. (**H**) MCF-7 and MCF-7/DDP or A549 and A549/5-Fu were treated with DDP or 5-Fu for 12 hours, followed by the LC-MS/MS analysis of R5P, S7P, or E4P. (**I** and **J**) MCF-7/DDP or A549/5-Fu cells were pretreated with NAC (5 mM) for 12 hours prior to treatment with DDP or 5-Fu. NADPH/NADP^+^ (**I**) and ROS levels (**J**) were analyzed. All experiments were repeated 3 times. *n* = 3. All error bars are mean ± SD. *P* values were calculated by 1-way ANOVA followed by Bonferroni’s test (**A**–**D**, **F**, and **G**). *P < 0.05, **P < 0.01, ***P < 0.001. NC, negative control; LC-MS/MS, liquid chromatography coupled with quadrupole time-of-flight mass spectrometry.

Increased ROS levels are a common cellular response to toxic xenobiotics (20). In line with this, we found that chemo drugs, including cisplatin (DDP), adriamycin (ADR), 5-fluorouracil (5-Fu), oxaliplatin (Oxa), gemcitabine (Gem), and paclitaxel (TAX), increased ROS levels in various human tumor cell lines (MCF-7, A549, HCT116, and SW1990) and mouse tumor cell lines (B16 and 4T-1) (Figure 1A). Notably, drug-resistant tumor cells (DRCs), generated from drug-induced parental tumor cells (MCF-7/DDP, MCF-7/ADR, A549/DDP, A549/5-Fu, HCT116/Oxa, SW1990/Gem, B16/TAX, and 4T-1/DDP) that are highly resistant to death by chemo drugs, displayed moderate increases in ROS, whereas parental cells, termed drug-sensitive tumor cells (DSCs), which are highly induced to die, showed strong increases in ROS (Figure 1A and Supplemental Figure 1A; supplemental material available online with this article; https://doi.org/10.1172/JCI170753DS1). Our observation indicated that ROS baseline levels in DRCs were higher than those in DSCs (Figure 1A). Hydrogen peroxide (H_2_O_2_) is the representative of ROS in cells due to its highest stability among the physiologically relevant ROS (20). Using red fluorescent sensor–expressing (HyPerRed) DRCs and DSCs to determine intracellular H_2_O_2_, we observed lower fluorescence intensity in DRCs, compared with DSCs, reflecting more ROS generated in DSCs in response to drug treatment (Supplemental Figure 1B). Quantitative detection showed that cytoplasmic H_2_O_2_ concentration was approximately 4 μM in DSCs and approximately 10 μM in DRCs. Adding moderate amounts of H_2_O_2_ to treat DSCs and DRCs led to fewer ROS increases in DRCs relative to DSCs (Supplemental Figure 1C), and under the condition of more than 20 μM H_2_O_2_ concentration in the cytoplasm, DRCs became sensitive to chemo drugs (Supplemental Figure 1D). These results suggest that DRCs are likely to evolve an intrinsic machinery to efficiently clear ROS during drug treatment. Similar to DRCs, drug-resistant tumor-repopulating cells (21–23) also had higher basic ROS levels than control bulk cells and moderately increased ROS levels upon drug treatment (Supplemental Figure 1E), suggesting that rapid clearance of ROS may be a shared mechanism to survive under drug treatment. NADPH plays a crucial role in ROS clearance. Coincidently, NADPH/NADP^+^ ratios were swiftly increased in the DRCs upon drug treatment (Figure 1B), which was not due to the catalytic effect of cytosolic malic enzymes (ME1, -2, and -3) or methylenetetrahydrofolate dehydrogenase 2 (MTHFD2) (Supplemental Figure 1, F–I). However, blocking PPP with 6-phosphogluconate dehydrogenase inhibitor 6-AN or siRNAs could disrupt the above increase in NADPH/NADP^+^ ratios, concomitant with a dramatic increase of ROS levels and the death of DRCs (Figure 1, C–G, and Supplemental Figure 1, J and K). In line with this, ultrahigh-performance liquid chromatography coupled with quadrupole time-of-flight mass spectrometry analysis showed that the PPP intermediate metabolites (R5P, S7P, and E4P) were much higher in the treated DRCs, compared with the treated DSCs (Figure 1H), suggesting that DRCs use PPP to provide NADPH. Intriguingly, such drug-induced NADPH production seemed to rely on high basic ROS levels, because pretreatment with the ROS scavenger N-acetyl-cysteine (NAC) or glutathione ethyl ester (GEE) impeded NADPH production and led to higher ROS levels in the drug-treated DRCs, concomitant with increased cell death (Figure 1, I and J, and Supplemental Figure 1, L–O). NADPH uses glutathione (GSH) to clear ROS (1, 20). In line with this, either knockdown of glutathione peroxidase 1 (GPX1) or inhibition of GSH synthesis led to excess ROS and DRC death upon drug treatment (Supplemental Figure 1, P–R); however, inhibition of superoxide dismutase (SOD) or catalase (CAT) did not affect the response of DRCs to chemo drugs (Supplemental Figure 1, S and T). Here, we also observed higher mitochondrial ROS levels and an increased activity of NADPH oxidase 2 (NOX2), which catalyzes ROS production, in DRCs relative to DSCs, suggesting that both the mitochondria and NOX2 enzymatic system contribute to the higher ROS levels in DRCs (Supplemental Figure 1, U–W). Together, these results suggest that DRCs use intrinsically high ROS levels for rapid NADPH production in response to chemo drug treatment.

### Glycogenolysis drives PPP in DRCs in response to drug molecules.

**Figure 2:**
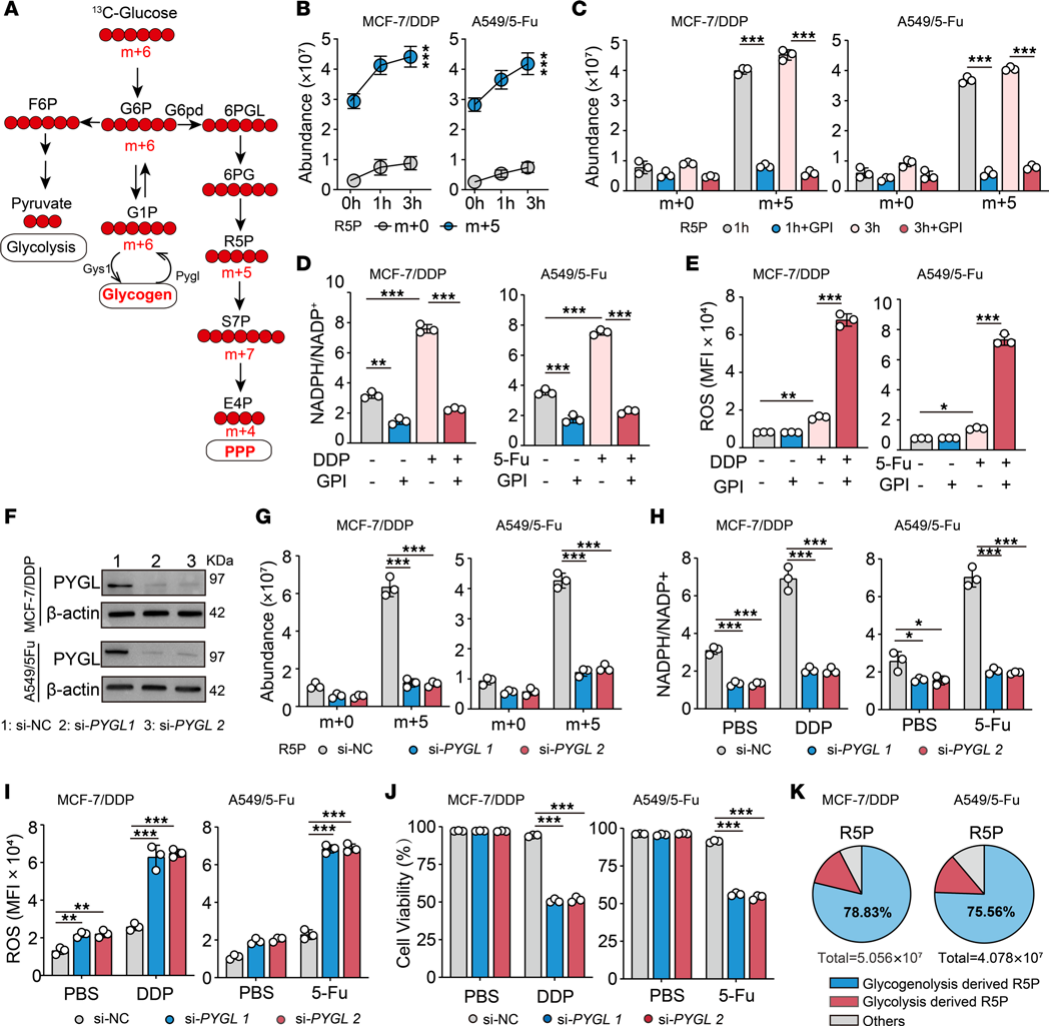
Glycogenolysis drives PPP in DRCs in response to drug molecules. (**A**) Overview of 3 glucose metabolic pathways: glycolysis, glycogen metabolism, and PPP are shown. (**B**) MCF-7/DDP or A549/5-Fu cells cultured in ^13^C-glucose were switched to ^12^C-glucose at the time of treatment with DDP or 5-Fu for 1 or 3 hours, and ^13^C-labeled R5P was detected by LC-MS/MS. (**C**) MCF-7/DDP or A549/5-Fu cells cultured in ^13^C-glucose were pretreated with GPI (50 μM) for 2 hours and switched to ^12^C-glucose at the time of chemo treatment. ^13^C-labeled R5P was detected by LC-MS/MS. (**D** and **E**) MCF-7/DDP or A549/5-Fu cells were treated with GPI for 2 hours prior to treatment with DDP or 5-Fu for 24 hours. NADPH/NADP^+^ (**D**) and ROS levels (**E**) were analyzed. (**F**) The expression of PYGL in MCF-7/DDP or A549/5-Fu cells transduced with si-NC or si-*PYGL* was analyzed by Western blot. (**G**) MCF-7/DDP or A549/5-Fu cells cultured in ^13^C-glucose transduced with si-NC or si-*PYGL* were switched to ^12^C-glucose for 4 hours of drug treatment, and ^13^C-labeled R5P or S7P was detected by LC-MS/MS. (**H** and **I**) MCF-7/DDP or A549/5-Fu cells transduced with si-NC or si-*PYGL* were treated with DDP or 5-Fu for 24 hours. NADPH/NADP^+^ (**H**) and ROS levels (**I**) were analyzed. (**J**) MCF-7/DDP and A549/5-Fu transduced with si-NC or si-*PYGL* were treated with DDP or 5-Fu for 48 hours. The cell viability was analyzed. (**K**) MCF-7/DDP or A549/5-Fu cells cultured in ^12^C-glucose pretreated with GPI for 2 hours were switched to ^13^C-glucose for 4 hours of drug treatment, and ^13^C-labeled R5P was detected by LC-MS/MS. All experiments were repeated 3 times. *n* = 3. All error bars are mean ± SD. *P* values were calculated by 2-tailed unpaired Student’s *t* test (**B**) or 1-way ANOVA followed by Bonferroni’s test (**C**–**E** and **G**–**J**). *P < 0.05, **P < 0.01, ***P < 0.001. G6pd, glucose-6-phosphate dehydrogenase; m+, number of carbon atoms labeled with ^13^C.

Next, we explored the mechanism by which PPP was triggered in the DRCs in response to drug treatment. To initiate PPP, G6P is first oxidized to ribulose 5-phosphate (Ru5P), followed by nonoxidative steps. Namely, Ru5P is converted either to ribose 5-phosphate (R5P) for nucleotide synthesis or to the R5P and xylulose 5-phosphate mixture, which leads to S7P and E4P as intermediates and glyceraldehyde 3-phosphate and fructose 6-phosphate (F6P) as end products (24, 25). Our previous reports have shown that glycogenolysis-generated G6P can be shunted to PPP (5, 6, 26). By culturing DRCs with uniformly labeled ^13^C-glucose for 10 days, which generated ^13^C-labeled glycogen with approximately 86% efficiency (Supplemental Figure 2A), and by switching to ^12^C-glucose culture medium in the presence of drugs, we found that drug treatment induced substantial ^13^C-labeled R5P in the DRCs (Figure 2, A and B). However, blocking glycogenolysis by glycogen phosphorylase inhibitor (GPI) disrupted carbon flow to R5P, concomitant with decreases in NADPH production, increases in ROS levels, and the death of DRCs (Figure 2, C–E, and Supplemental Figure 2B). DRCs mainly expressed the liver form of glycogen phosphorylase (PYGL) rather than the brain or muscle form in response to drug treatment (Supplemental Figure 2C). Consistently, the use of PYGL siRNA also led to similar results as GPI (Figure 2, F–J); the blockade of glycogen synthesis also generated consistent results (Supplemental Figure 2, D–F), suggesting that glycogenolysis-derived G6P is channeled into the PPP. In addition, the blockade of glycogenolysis showed that little ^13^C-glucose–derived G6P flowed to PPP in drug-treated DRCs, as evaluated by the abundance of m+5 R5P or m+7 S7P (Figure 2K and Supplemental Figure 2G). Together, these results suggest that glycogenolysis drives PPP in the DRCs in response to drug treatment.

### DRCs use AHR to promote glycogenolysis.

**Figure 3:**
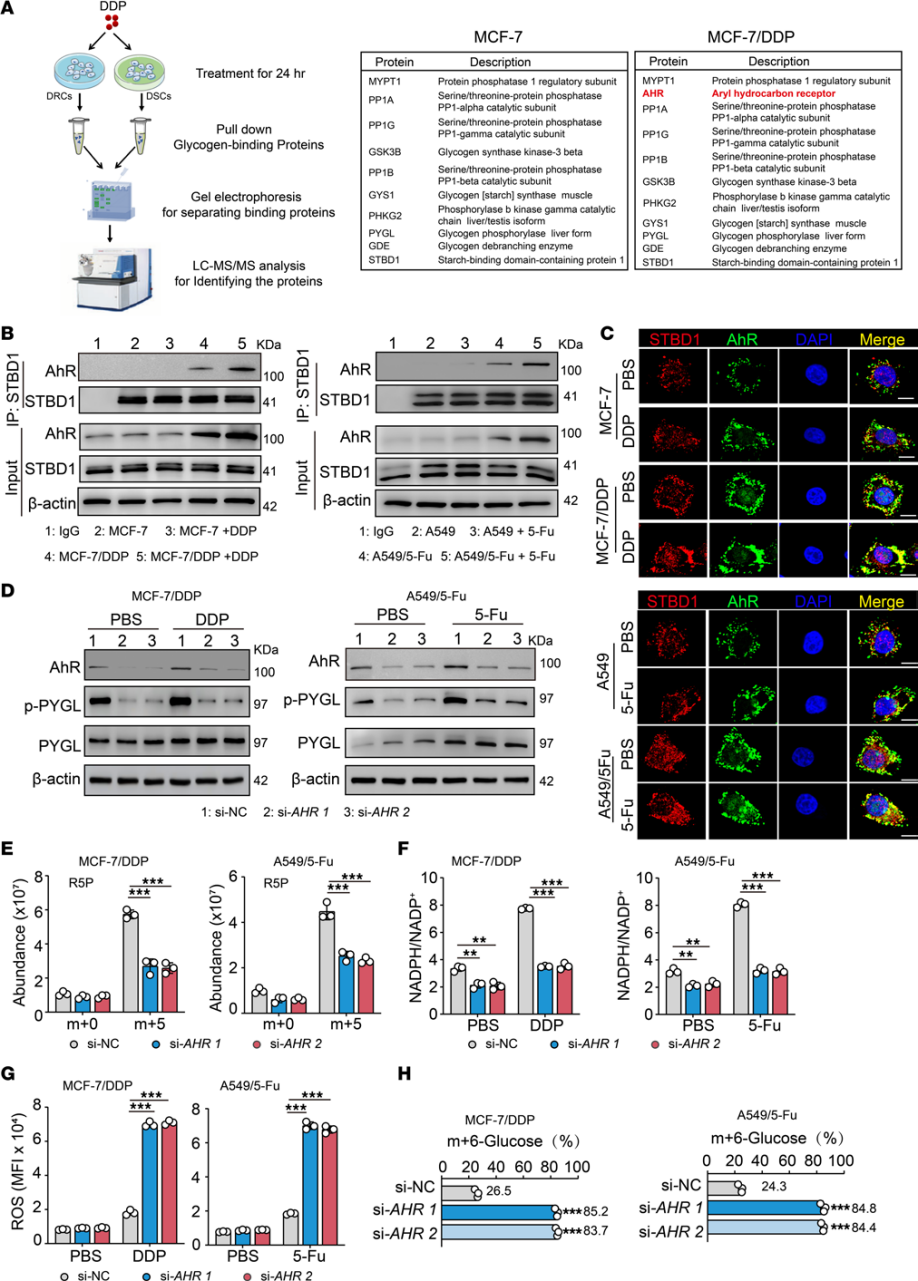
DRCs use AHR to promote glycogenolysis. (**A**) MCF-7 or MCF-7/DDP cells were treated with DDP for 24 hours. Cell lysates were immunoprecipitated with anti-STBD1 for MS. Identified proteins are listed. (**B**) Immunoblot of immunoprecipitations of STBD1 in lysates from cells treated with DDP or 5-Fu for 24 hours. (**C**) Cells were treated with DDP or 5-Fu for 24 hours. The location of AHR (green) and STBD1 (red) was observed under confocal microscope. Scale bar, 10 μm. (**D**) MCF-7/DDP or A549/5-Fu cells transduced with si-NC or si-AHR were treated with DDP or 5-Fu for 24 hours. Phosphorylated (phospho-) PYGL or PYGL was analyzed by Western blot. (**E**) MCF-7/DDP or A549/5-Fu cells cultured in ^13^C-glucose transduced with si-NC or si-*AHR* were switched to ^12^C-glucose for 4 hours of drug treatment, and ^13^C-labeled R5P was detected by LC-MS/MS. (**F** and **G**) MCF-7/DDP or A549/5-Fu cells transduced with si-NC or si-*AHR* were treated with DDP or 5-Fu for 24 hours. NADPH/NADP^+^ (**F**) and ROS levels (**G**) were analyzed. (**H**) MCF-7/DDP or A549/5-Fu cells cultured in ^13^C-glucose for 10 days were transduced with si-NC or si-*AHR*, then treated with DDP or 5-Fu for 8 hours, followed by treatment with hydrochloric acid, leading to the degradation of polymer glycogen into monomer glucose. The amount of released ^13^C-labeled glucose (m+6) was determined by LC-MS/MS. The effect of blocked glycogen degradation by AHR knockdown is evaluated by the amount of m+6 glucose, with the formula ([si-AHR1% + si-AHR2%]/2) – si-NC%. All error bars are mean ± SD. *P* values were calculated by 1-way ANOVA followed by Bonferroni’s test (**E**–**H**); *n* = 3; **P < 0.01, ***P < 0.001.

Next, we investigated the molecular mechanism by which glycogenolysis was promoted in DRCs in response to drug treatment. Glycogen is assembled with glycogen metabolic enzymes to form a carbohydrate-protein complex (27–29). Pulling down the complex from drug-treated DSCs and DRCs with anti–starch-binding domain containing protein 1 (anti-STBD1) antibody, we analyzed glycogen-associated proteins in the precipitate by MS. As expected, glycogen synthase (GYS), glycogen phosphorylase (GP), debranching enzyme, phosphorylase kinase (PhK), PP1c, and PP1 regulatory subunits (GM, GL, PTG) were included in the complex. Surprisingly, AHR, a sensor of xenobiotics, was also included in the glycogen complex of the DRCs (Figure 3A and Supplemental Figure 3A), which was further verified by Western blot and fluorescence microscopy (Figure 3, B and C). Notably, unlike DRCs, DSCs expressed much lower levels of AHR, which seemed unlikely to be induced by drugs (Figure 3, B and C). Knocking down AHR with siRNAs or by using its inhibitor, StemRegenin 1 (SR1), resulted in the abrogation of glycogenolysis and PPP in the drug-treated DRCs, concomitant with increased ROS levels (Figure 3, D–G, and Supplemental Figure 3, B–E), suggesting that AHR is involved in glycogenolysis in drug-treated DRCs. Using ^13^C-glucose–cultured DRCs, we further traced the effect of AHR on glycogen degradation. We found that after 8-hour drug treatment, 57.95% and 47% or 60.3% and 45.9% glycogen degradation was blocked by the AHR knockdown and inhibitor, respectively (Figure 3H and Supplemental Figure 3F). AHR has been reported to promote cancer stem cell (CSC) reprogramming (30, 31). By determining CSC markers (aldehyde dehydrogenase, CD133, CD90, organic cation/carnitine transporter 4 [OCT4], SRY box transcription factor 2, and β-catenin) and epithelial-mesenchymal transition markers (E-cadherin, vimentin, and SNAIL), we found that DRCs and DSCs displayed similar expression patterns (Supplemental Figure 3, G–O). Together, these results suggest that AHR is required for glycogenolysis in DRCs in response to drug treatment.

### Cysteine sulfenylation licenses AHR to bind to glycogen particles.

**Figure 4:**
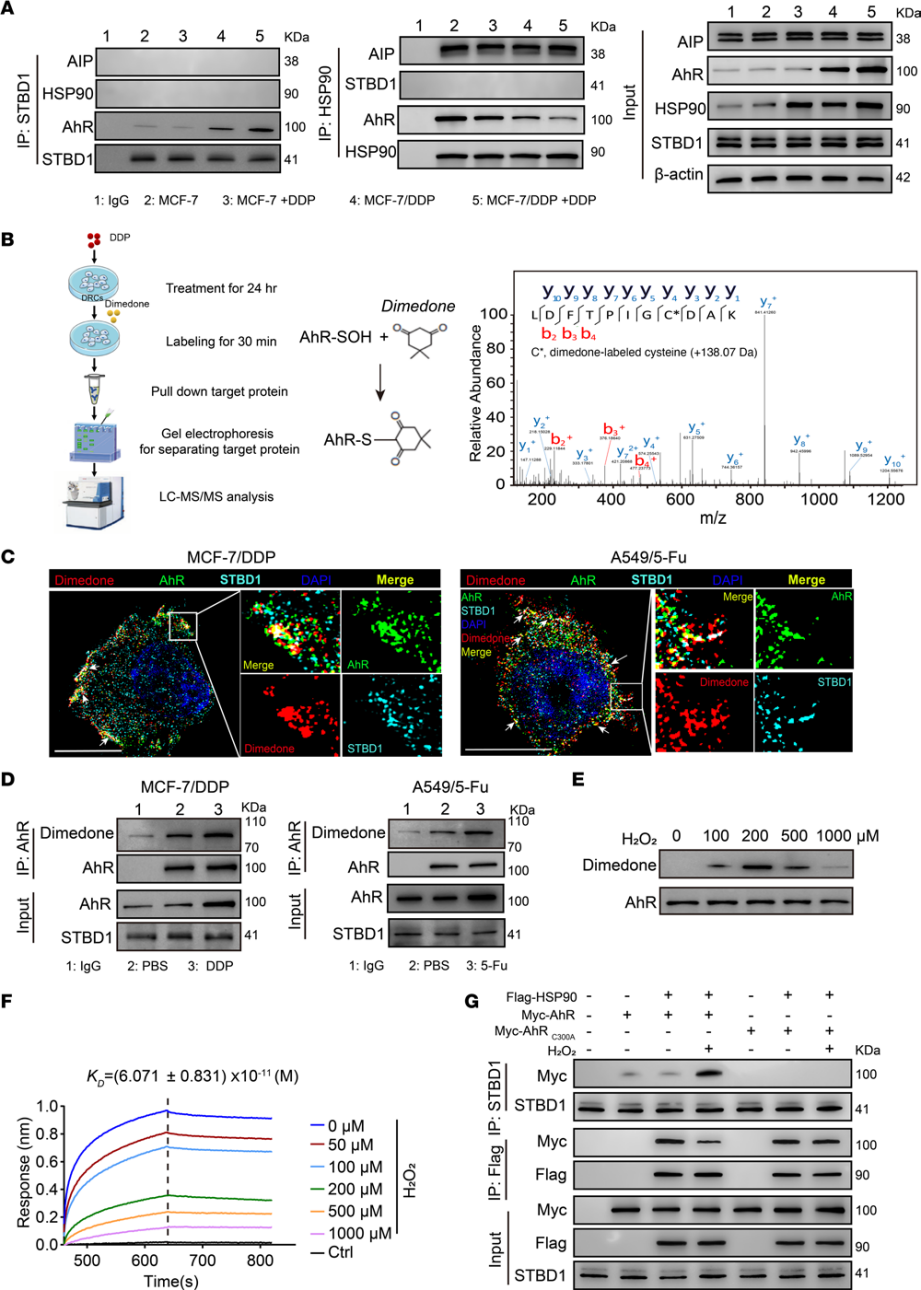
Cysteine sulfenylation licenses AHR to bind to glycogen particles. (**A**) Immunoblot of immunoprecipitations of STBD1 or HSP90 in lysates from MCF-7 or MCF-7/DDP cells treated with DDP for 24 hours. (**B**) Strategy for detecting sulfenic acid modification of AHR with dimedone. LC-MS/MS analysis of dimedone-labeled AHR. Analysis of the y-ions indicates the formation of dimedone adduct (+138.07 Da). (**C**) MCF-7/DDP or A549/5-Fu cells were treated with DDP or 5-Fu for 24 hours. The location of AHR (green), dimedone (red), and STBD1 (cyan) was observed under super-resolution microscope. The arrows indicate the colocation of the sulfenylated AHR and STBD1. Scale bars, 10 μm. (**D**) Separated glycogen-enriched pellets from MCF-7/DDP or A549/5-Fu cells treated with DDP or 5-Fu for 24 hours were resuspended in IP buffer, and immunoblot with dimedone of immunoprecipitations of AHR was analyzed. (**E**) Purified Flag-tagged (Flag-AHR) protein was treated with different doses of H_2_O_2_ for 30 minutes. Sulfenic acid modification of dimedone-labeled AHR was analyzed by Western blot. (**F**) Purified Flag-AHR protein was treated with different doses of H_2_O_2_ for 30 minutes. The binding between modified AHR and HSP90 was measured by biolayer interferometry (BLI). (**G**) HEK293T cells transfected with the indicated combinations of Flag-HSP90, Myc-AHR, or Myc-AHR(C300A) were treated with H_2_O_2_ for 30 minutes. Immunoprecipitations of Flag or STBD1 were analyzed by Western blot.

AHR exists in the cytoplasm of intact cells by forming a complex with 2 heat shock protein 90 (HSP90) chaperones, AHR-interacting protein (AIP) and cochaperone p23 (11). Notably, despite the binding of AHR to glycogen, HSP90 was not associated with the glycogen complex in drug-treated DRCs, as evidenced by the pulldown with anti-STBD1 or anti-HSP90 (Figure 4A and Supplemental Figure 4A). Meanwhile, we found that the binding of AHR and HSP90 was reduced in treated DRCs relative to untreated ones. Similar to HSP90, AIP was also not found in the glycogen particles, and its binding to AHR was reduced in the drug-treated DRCs (Figure 4A and Supplemental Figure 4A). These results prompted us to speculate that a switch of AHR from the HSP90 complex to glycogen complex occurs in response to drug treatment, which might be due to the conformational change. Given the increased ROS and the ability to modify cysteine residues of proteins (32, 33), we assumed that ROS mediated cysteine sulfenylation of AHR by conversion of thiolate group (S-) to sulfenic acid (SOH). Using dimedone as a probe (34, 35), we found that Cys 300 of AHR was sulfenylated in drug-treated DRCs by LC-MS/MS (Figure 4B and Supplemental Figure 4B). Further fluorescence staining and Western blot analyses showed that sulfenylated AHR was indeed present in the glycogen particles (Figure 4, C and D). In line with this, increases in cytoplasmic H_2_O_2_ concentrations (< 20 μM) resulted in increased AHR sulfenylation (Supplemental Figure 4C). In addition, using H_2_O_2_ to treat the AHR protein directly in vitro, we observed an overt sulfenylation of AHR (Figure 4E and Supplemental Figure 4D), and this sulfenylated AHR lost its ability to bind to HSP90 (Figure 4F). In line with these results, we found that the mutation of Cys 300 to alanine prevented the binding of AHR to glycogen particles but regained the ability to bind HSP90 upon H_2_O_2_ treatment (Figure 4G). To clarify how drug molecules increase ROS levels for AHR Cys 300 sulfenylation, we focused on P450, a main detoxifying enzymatic system in cells (18, 36). Using a pan-P450 inhibitor to treat DRCs and DSCs in the presence of chemo drugs, we found that the effect of drug molecules on ROS content was disrupted in both cells (Supplemental Figure 4E). Together, these results suggest that cysteine sulfenylation licenses the binding of AHR to glycogen particles for glycogenolysis.

### Sulfenylated AHR promotes GP activity by competitively binding PPP1R3C.

**Figure 5:**
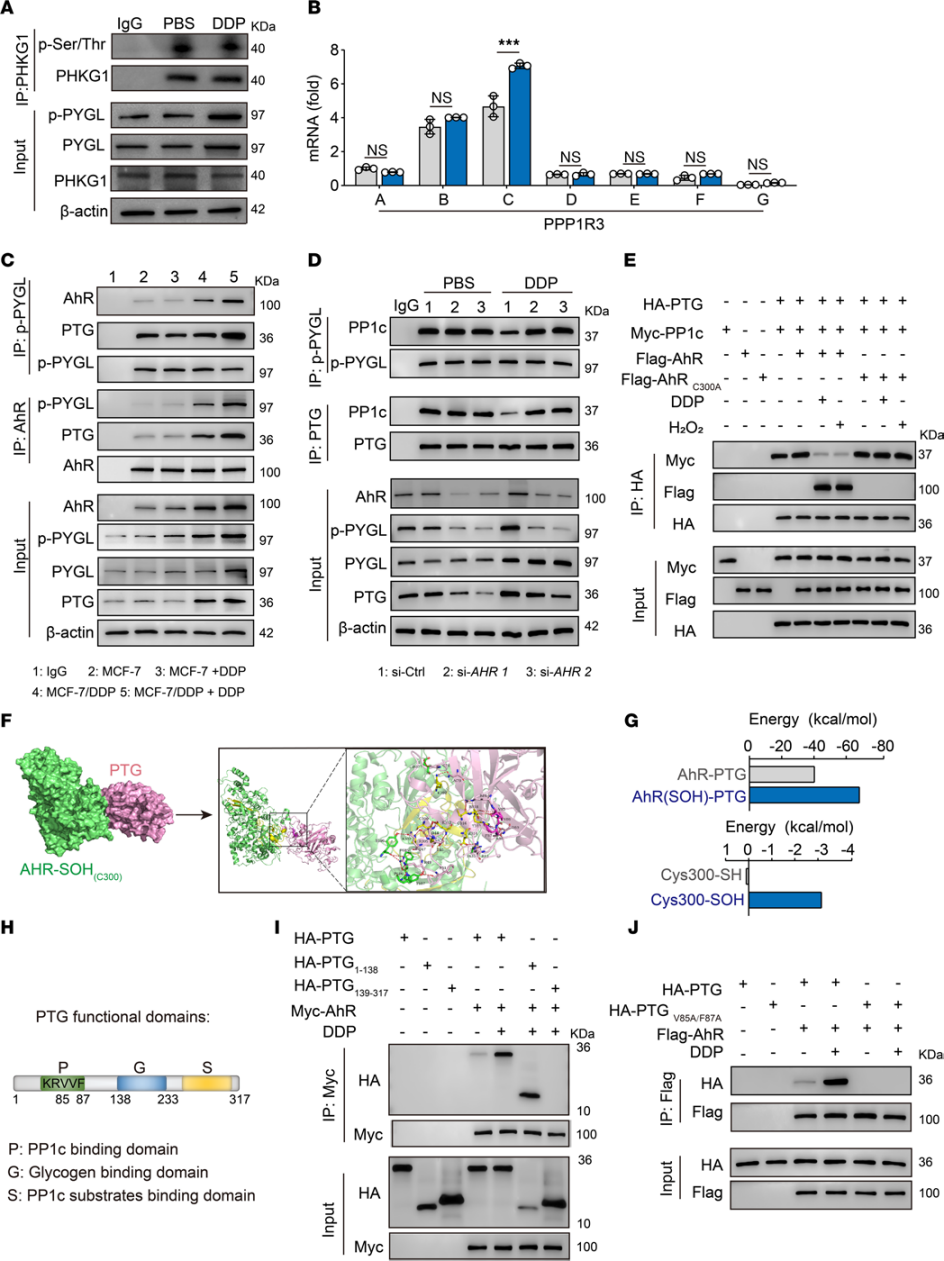
Sulfenylated AHR promotes GP activity by competitively binding PTG. (**A**) DRCs were treated with DDP for 24 hours. Phospho-PHKG1, phospho-PYGL, PYGL, and PHKG1 were analyzed by Western blot. (**B**) DRCs were treated with DDP for 24 hours. PPP1R3A–G expression was determined by real-time PCR. (**C**) Immunoblot of immunoprecipitations of p-PYGL or AHR in lysates from MCF-7 or MCF-7/DDP cells treated with DDP for 24 hours. (**D**) Immunoblot of immunoprecipitations of p-PYGL or PTG in lysates from DRCs transfected with si-Ctrl or si-*AHR* treated with DDP for 24 hours. (**E**) HEK293T cells transfected with the indicated combinations of HA-PTG, Myc-PP1c, Flag-AHR, or Flag-AHR(C300A) were treated with DDP (20 μM) for 12 hours or H_2_O_2_ (200 μM) for 30 minutes. Immunoprecipitations of HA were analyzed by Western blot. (**F**) The 3D surface binding model of PTG with AHR-(SOH). AHR-(SOH) is colored with green, and the 240–330 residues in AHR are colored in yellow. PTG is colored with pink, and the 85–87 residues in AHR are colored in magenta. The red dashes represent hydrogen bond interactions. The blue dashes represent salt bridges. (**G**) The average binding free energy of AHR(SOH)-PTG and AHR-PTG and energy decomposition in Cys 300 of AHR(SOH)-PTG and AHR(SH)-PTG. (**H**) Schematic diagram of PTG functional domains. (**I**) HEK293T cells transfected with the indicated combinations of HA-PTG, Myc-AHR, HA-PTG_1-138_, and HA-PTG_139-317_ were treated with DDP (20 μM) for 12 hours. Immunoprecipitations of Myc were analyzed by Western blot. (**J**) HEK293T cells transfected with the indicated combinations of HA-PTG, Flag-AHR, or HA-PTG_V85A/F87A_ were treated with DDP (20 μM) for 12 hours. Immunoblot of immunoprecipitations of Flag was analyzed by Western blot. All error bars are mean ± SD. *P* values were calculated by 2-tailed unpaired Student’s *t* test (**B**); *n* = 3; ***P < 0.001.

Next, we investigated how oxidized AHR promotes glycogenolysis. Enhanced glycogenolysis requires increased PYGL activity. Phospho-PhK is the major kinase, which activates PYGL by phosphorylating Ser 14 (37). We found that PYGL phosphorylation was markedly elevated in drug-treated DRCs, while PhK activity was not altered (Figure 5A and Supplemental Figure 5A), suggesting that a dephosphorylation mechanism might be involved in PYGL regulation in DRCs in response to drug treatment. The dephosphorylation of PYGL is mediated by PP1c in conjunction with a regulatory subunit of the PPP1R3 family, including 7 members (PPP1R3A–G), which are characterized by a binding site for PP1c, a binding domain for glycogen, and binding PP1c substrates (38). Notably, the expression of PPP1R3C, also known as PTG (39), was markedly upregulated, but the expression of other PPP1R3 members in DRCs was not (Figure 5B and Supplemental Figure 5B), prompting us to hypothesize that sulfenylated AHR interfered with PP1c-mediated dephosphorylation of PYGL. Using an AHR antibody to pull down lysates from DRCs, we found that both PTG and phospho-PYGL (p-PYGL) could be detected in the immunoprecipitation (Figure 5C and Supplemental Figure 5C). Similarly, PTG and AHR coprecipitated with the p-PYGL antibody. The binding of p-PYGL and PTG, and the binding of p-PYGL and AHR, could be enhanced by drug treatment (Figure 5C and Supplemental Figure 5C). In contrast with AHR-binding PTG, the binding of PP1c to PTG was reduced by drug treatment, which, however, could be rescued by AHR knockdown (Figure 5D and Supplemental Figure 5D), suggesting that sulfenylated AHR competes with PP1c for binding to PTG. To further verify this, we introduced HA-PTG, Myc-PP1c, and Flag-AHR into HEK293T cells and found that either H_2_O_2_ or drug treatment promoted the binding of PTG with AHR but reduced the binding of PTG with PP1c. However, when Flag-mutated AHR (Cys 300 to Ala, C300A) was introduced, the binding of PTG with PP1c was rescued (Figure 5E). In addition, analyzing AHR-PTG interaction by molecular docking and molecular dynamics simulation, we found that AHR used the sulfenylation site (C300-SOH) to bind to PP1c-binding domain of PTG (Figure 5, F and G, and Supplemental Figure 5, E and F). Moreover, we constructed mutated *AHR*(C300A)- and unmutated *AHR*(C300)-overexpressing DRCs with the knockout of endogenous AHR. Using chemo drugs to treat *AHR*-KO, *AHR*(C300), or *AHR*(C300A) DRCs, we found that excess ROS was present in *AHR*-KO and *AHR*(C300A) DRCs with the loss of the drug-resistant ability. Concomitantly, *AHR*(C300A) neither bound to PTG nor increased glycogenolysis and NADPH (Supplemental Figure 5, G–J). Moreover, using the PTG fragment with the PP1c-, glycogen-, or PP1 substrate–binding domain (27, 40), we found that sulfenylated AHR bound only the fragment with PP1c-binding domain (Figure 5, H and I). Given the RVxF motif in the PP1c-binding domain, we also constructed PTG double mutations (V85A and F87A). We found that dual mutations blocked the binding of PTG to AHR (Figure 5J). Thus, sulfenylated AHR may compete with PP1c to bind the RVxF motif of PTG. Sulfenylation can be modified by GSH and lead to *S*-glutathionylation, another reversible cysteine residue–modifying form (41). Western blot showed that AHR *S*-glutathionylation was present in DRCs and was enhanced by drug treatment (Supplemental Figure 5K); however, LC-MS/MS analysis of PTG-bound AHR did not show *S*-glutathionylation on Cys 300 in drug-treated DRCs. In addition, overexpression or knockdown of GRX1, the enzyme that catalyzes deglutathionylation, did not affect the binding of AHR to PTG, concomitant with unaltered PYGL phosphorylation, G6PD enzymatic activity, and NADPH production (Supplemental Figure 5, L–O). Together, these results suggest that sulfenylated AHR inhibits PP1c from dephosphorylating PYGL by binding to PTG.

### Sulfenylated AHR–regulated glycogenolysis promotes drug resistance in vivo.

**Figure 6:**
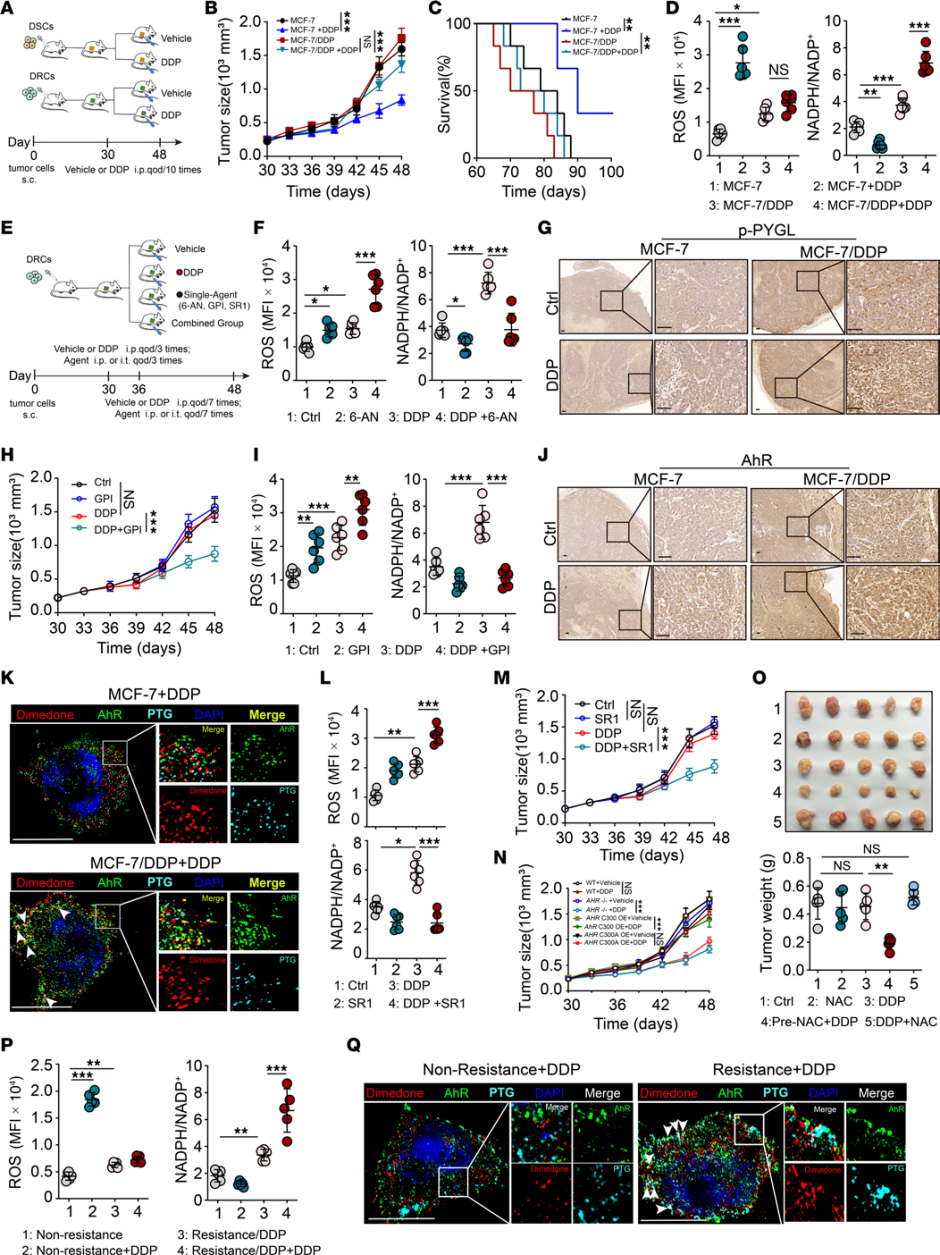
Sulfenylated AHR–regulated glycogenolysis promotes drug resistance in vivo. (**A**) Schematic of experimental design. (**B**–**D**) Tumor-bearing mice were administrated with DDP. The tumor growth (**B**) and mouse survival (**C**) were monitored. ROS and NADPH/NADP^+^ (**D**) in isolated tumor cells were analyzed. (**E**) Schematic of experimental design for mouse treatment. (**F**) DDP-treated tumor-bearing mice were administrated with 6-AN. ROS and NADPH/NADP^+^ were analyzed. (**G**) Immunohistochemical staining of p-PYGL from the sections of tumor tissues in tumor-bearing mice. Scale bar, 50 μm. (**H** and **I**) DDP-treated tumor-bearing mice were administrated with GPI, tumor growth was monitored, and ROS or NADPH/NADP^+^ were analyzed. (**J**) Immunohistochemical staining of AHR from the sections of tumor tissues in tumor-bearing mice. Scale bar, 50 μm. (**K**) Isolated tumor cells were labeled with dimedone. AHR (green) and PTG (cyan) were observed under super-resolution microscope. The arrows indicate the colocation of the sulfenylated AHR and PTG. Scale bars, 10 μm. (**L** and **M**) DDP-treated tumor-bearing mice were administrated with SR1. ROS and NADPH/NADP^+^ were analyzed and tumor growth was monitored. (**N**) *AHR*^–/–^ and *AHR*(C300A)- or *AHR*(C300)-overexpressing (OE) DRCs were inoculated into NSG mice. Tumor growth of treated tumor-bearing mice was monitored. (**O**) DDP-treated tumor-bearing mice were administrated with NAC 5 times or pretreated with NAC for 3 days before being administrated with DDP. Tumor size was presented photographically (top) or by weight (bottom). Scale bars, 1 cm. (**P** and **Q**) DDP-resistant MMTV-PyMT mice were administrated with DDP 3 times. ROS levels and NADPH/NADP^+^ in isolated tumor cells were analyzed. AHR (green) and PTG (cyan) were observed. The arrows indicate the colocation of the sulfenylated AHR and PTG. Scale bars, 10 μm. **A**–**M**, *n* = 6 mice; **N**–**Q**, *n* = 5 mice. All error bars are mean ± SD. *P* values were calculated by 1-way ANOVA followed by Bonferroni’s test (**B**, **D**, **F**, **H**, **I**, and **L**–**P**), and log-rank test (**C**); *P < 0.05, **P < 0.01, ***P < 0.001. qod, every other day.

Next, we investigated whether the above in vitro findings could be verified in vivo. To this end, we implanted DRCs (MCF-7/DDP or A549/5-Fu) into NOD/SCID IL-2Rγ–null (NSG) mice, followed by treatment with DDP or 5-Fu. We found that compared with MCF-7 or A549 DSCs, DRCs formed larger tumors and shortened the survival of the mice (Figure 6, A–C, and Supplemental Figure 6, A and B). By dissecting tumor cells in the treated mice, we found that following drug treatment, DRCs had lower ROS and higher NADPH levels as well as increased content of PPP intermediate metabolites such as R5P, S7P, and E4P, compared with the corresponding DSCs (Figure 6D and Supplemental Figure 6, C–E). However, such alterations could be reversed by injection of 6-AN, which blocked the PPP of the cells (Figure 6, E and F, and Supplemental Figure 6, F and G). Consistently, PPP inhibition had a marginal effect on the treatment outcome in the DSC group but enhanced the treatment efficacy in the DRC group (Supplemental Figure 6H). In addition, we found that p-PYGL levels were higher in DRCs than in DSCs both before and after drug treatment (Figure 6G and Supplemental Figure 6I). Either knockout or inhibition of PYGL, which did not affect the growth of DRCs in the mice (Supplemental Figure 6J), resulted in increased cellular sensitivity to the drugs, concomitant with increases in ROS levels and decreases in NADPH/NADP^+^ ratios (Figure 6, H and I, and Supplemental Figure 6, K–N), suggesting that glycogenolysis is an upstream event of PPP in drug-resistant tumors. We then verified that sulfenylated AHR regulated PYGL activity in mice. We found that AHR expression was higher in DRCs than in the corresponding DSCs (Figure 6J and Supplemental Figure 6O), concomitant with the increased colocalization of sulfenylated AHR and PTG in the glycogen complex (Figure 6K and Supplemental Figure 6P). Moreover, we found that AHR inhibition or knockout led to decreases in p-PYGL and NADPH/NADP^+^ ratios and increased ROS levels in the DRCs, as well as smaller tumor sizes and prolonged survival of the mice (Figure 6, L and M, and Supplemental Figure 7, A–F). Moreover, inoculating *AHR*(C300A)- or *AHR*(C300)-overexpressing DRCs into NSG mice, we found that C300A mutation inhibited tumor growth (Figure 6N). In addition, inoculating Tet-inducible *AHR* shRNA DRCs into NSG mice also resulted in the inhibition of tumor growth (Supplemental Figure 7, G and H). Given the role of ROS in AHR regulating PYGL, we pretreated tumor-bearing mice with antioxidants including NAC or GEE before drug treatment. Intriguingly, we found that the NAC or GEE pretreatment effectively increased the inhibition of tumor growth; however, the synchronous combination of NAC or GEE with drugs did not increase the inhibition of tumor growth (Figure 6O and Supplemental Figure 7, I and J). As expected, NAC pretreatment did not improve the inhibition of tumor growth in *AHR*(C300A)-mutated tumor-bearing mice (Supplemental Figure 7K). In addition to the above tumor cell–implanted models, we also tested the MMTV-PyMT spontaneous breast tumor model. As shown in Supplemental Figure 7, L–N, DDP resistance was established in vivo. Consistently, we found that ROS and NADPH levels were lower and higher, respectively, in the resistant tumor cells, compared with the control unresistant cells, which were further enhanced by DDP treatment, concomitant with AHR sulfenylation and colocalization with PTG of the glycogen complex (Figure 6, P and Q). Together, these results suggested that sulfenylated AHR–regulated glycogenolysis promotes drug resistance in tumor cells in vivo.

### AHR glycogenolysis occurs in patients with chemoresistant cancer.

**Figure 7:**
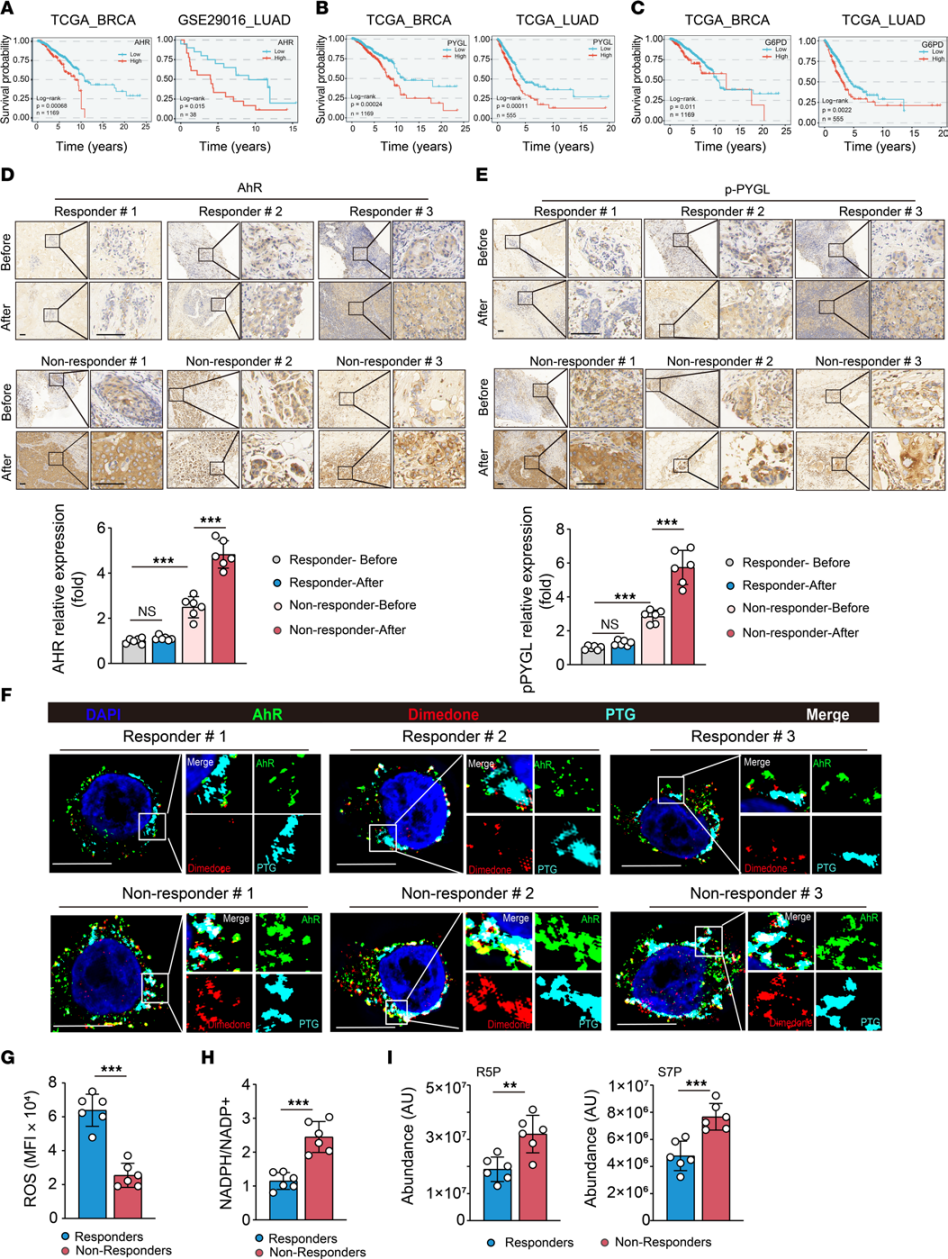
AHR glycogenolysis occurs in patients with chemoresistant cancer. (**A**–**C**) Overall survival on the basis of AHR level in people with breast cancer (*n* = 1,669) or lung cancer (*n* = 38) (**A**), PYGL level in people with breast cancer (*n* = 1,669) or lung cancer (*n* = 555) (**B**), and G6PD level in people with breast cancer (*n* = 1,669) or lung cancer (*n* = 555) (**C**). (**D** and **E**) The tissue sections from patients with breast cancer including responders or nonresponders before and after chemotherapy were immunohistochemically stained with anti-AHR antibody (**D**) and anti–p-PYGL antibody (**E**). Scale bars, 50 μm. (*n* = 6 per group.) (**F**) Isolated primary tumor cells from fresh breast cancer tissues including responders and nonresponders after chemotherapy were treated with dimedone (5 mM) for 30 minutes. The location of AHR (green), dimedone (red), and PTG (cyan) in the isolated tumor cells of tumor tissues was observed under super-resolution microscope. Scale bars, 10 μm. (*n* = 6 per group.) (**G**–**I**) ROS levels (**G**), the ratio of NADPH/NADP^+^ (**H**), and R5P and S7P (**I**) in isolated primary tumor cells from fresh breast cancer tissues including responders and nonresponders after chemotherapy were analyzed (*n* = 6 per group). All error bars are mean ± SD. *P* values were calculated by 1-way ANOVA followed by Bonferroni’s test (**D** and **E**), 2-tailed unpaired Student’s *t* test (**G**–**I**), and log-rank test (**A**–**C**). **P < 0.01, ***P < 0.001.

Finally, we sought to validate our findings by using clinical patient samples. In The Cancer Genome Atlas (TCGA) and Genomic Spatial Event (GSE) databases, we found that a higher expression of AHR was correlated with a worse prognosis in patients with several cancer types, including breast cancer, lung cancer, glioma, and pancreatic adenocarcinoma (Figure 7A and Supplemental Figure 8A). Meanwhile, the PYGL and G6PD levels in the primary tumors, including breast cancer, lung adenocarcinoma, and colon cancer, were positively correlated with poor patient survival (Figure 7, B and C, and Supplemental Figure 8, B and C). Based on these correlations, we further investigated whether the AHR-PYGL machinery was active in clinical nonresponders to chemotherapy. Breast and lung cancer tissues were obtained by needle biopsy before chemotherapy, whereas after chemotherapy, tumor tissues were obtained by surgery. The chemotherapy responders and nonresponders were evaluated according to the Response Evaluation Criteria in Solid Tumors. We found that AHR and p-PYGL were more highly expressed in tumor tissues from nonresponders than in responders after chemotherapy (Figure 7, D and E, and Supplemental Figure 8, D and E). Moreover, we isolated primary tumor cells from fresh breast and lung cancer tissues (6 responders and 6 nonresponders, each tumor type). By staining the cells, we found that sulfenylated AHR was colocated with PTG of glycogen complex in nonresponders after chemotherapy (Figure 7F and Supplemental Figure 8F). In line with this, ROS levels decreased, along with increased NADPH/NADP^+^ ratios and R5P/S7P levels, in the cells of nonresponders (Figure 7, G–I, and Supplemental Figure 8, G–I). Together, these results implied that sulfenylated AHR–regulated glycogenolysis might contribute to chemoresistance in patients with cancer.

## Discussion

Chemotherapy is used in most patients with cancer; however, intrinsic or acquired drug resistance can cause treatment failure (42). The upregulation of multidrug transporters, insensitivity to drug-induced apoptosis, and induction of drug detoxification are the most common reasons for the acquisition of drug resistance, whereas tumor cell stemness and microenvironments are responsible for the intrinsic resistance (43, 44). However, mechanisms underlying drug resistance are not completely understood. In this study, we reveal an ROS clearance–based molecular pathway to mediation of drug resistance. Upon entering tumor cells, drug molecules induce considerable ROS production, which triggers the AHR/glycogen phosphorylase/glycogenolysis/PPP pathway, leading to NADPH generation and subsequent ROS clearance as well as drug resistance.

Glycogens are conventionally understood to store and supply energy to the liver and muscles. Mounting evidence indicates that glycogen also plays critical roles in cell differentiation, signaling, stemness, and redox regulation under various physiological and pathophysiological conditions. It has been highlighted that the gluconeogenesis/glycogenesis/glycogenolysis/PPP metabolic chain plays an essential role in redox homeostasis (45). This notion is further strengthened by the present study, which shows that glycogen metabolism is an important player in cancer drug resistance via ROS regulation. Although our studies consistently revealed that glycogen-derived G6P can be shunted to PPP, glycogenolysis-derived G6P faces a fluxing choice between glycolysis and PPP (5, 6, 26). The underlying mechanism has not been explored to our knowledge, but it is worthwhile to determine what kind of situation or signal(s) induces cells to allow the shunt of glycogenolysis-derived G6P to PPP. Notwithstanding this, regulation of glycogen degradation has been widely investigated, especially for GP, the rate-limiting enzyme of glycogenolysis. GP is regulated by dephosphorylation (inactive form) and phosphorylation (active form). Glucagon signaling activates PhK via PKA, thereby phosphorylating GP (37). Although serine phosphorylation is conventionally appreciated to contribute to GP activation, tyrosine phosphorylation may also induce GP activation (26). On the other hand, GP dephosphorylation is commonly mediated by PP1c, which can be recruited to the glycogen complex upon binding the regulatory subunit (39). In this study, we unexpectedly found that GP dephosphorylation was regulated by AHR.

Given its activation by a variety of ligands, AHR is thought to be an exposome receptor (14), which not only recognizes environmental stimuli, such as toxins, light, heat, and cold, but also responds to immune factors, endogenous metabolites, and cell-derived harmful molecules. In the present study, we demonstrated that AHR can act as an ROS sensor in response to intracellular ROS stress. Structurally, the AHR peptide chain contains numerous cysteine residues (18 in human AHR) (46). We found that C300 was oxidized to SOH. This sulfenylated AHR then dissociated from the HSP90 complex and was recruited to the glycogen complex, where AHR competitively bound to the PP1 regulatory subunit PTG, blocking the binding of PP1c to PTG and subsequent GP dephosphorylation, thus promoting glycogenolysis. As a result, glycogen-derived G6P was shunted to the PPP, thus supplying NADPH to clear ROS for the survival of drug-treated tumor cells. Previously, we demonstrated that both IFN-β and IFN-γ are able to induce highly tumorigenic cells to enter a dormant state by activating AHR (30, 31). Besides IFNs, we found that IL-2 signaling can result in AHR activation via the STAT5/tryptophan hydroxylase 1 pathway, thus inducing CD8^+^ T cells into exhaustion (47). Taken together, these findings suggest that AHR has an unusual ability to exert a lifesaving effect, regardless of the AHR-induced differences in dormancy, exhaustion, and ROS clearance. In fact, AHR was originally discovered in the detoxification of 2,3,7,8-tetrachlorodibenzo-p-dioxin, which is a typical function that allows cell survival (48). Therefore, we propose here that AHR is not only an exposome receptor, but also more importantly, the guardian of cells to sense various cellular stresses and maintain homeostasis for cell survival. This may be a consequence of cell evolution. Ancestral cells face numerous extracellular and intracellular toxins and/or stresses, but the small genome size does not allow the cell to express enough protein molecules to respond individually. In this case, AHR was selected as a representative response to various stresses to improve the survival of cells.

In summary, the data in this study clearly show that AHR, by virtue of its sulfenylation, acquires the ability to bind PTG of the glycogen complex, leading to PP1c losing GP dephosphorylation, thus promoting glycogenolysis and subsequent PPP in DRCs. The resultant NADPH, in turn, guarantees ROS clearance and allows cells to survive. This study opens an avenue for understanding of AHR by its sensing ROS and maintaining redox homeostasis of cells, thus providing strategies to reverse tumor drug resistance.

## Methods

### Animals and cell lines.

Six-week-old female NSG mice and spontaneous breast tumor model MMTV-PyMT mice were purchased from the Center of Medical Experimental Animals of the CAMS (Beijing, China). These animals were maintained in the Animal Facilities of the CAMS under pathogen-free conditions.

Murine B16 melanoma and 4T-1 breast cancer and human MCF-7 breast cancer, A549 lung cancer, HCT116 colon cancer, and SW1990 pancreatic carcinoma cell lines were purchased from the China Center for Type Culture Collection (Beijing, China). B16, MCF-7, HCT116, and SW1990 cell lines were cultured in a RPMI-1640–based medium, or 4T-1 and A549 cell lines were cultured in a DMEM-based medium supplemented with 10% FBS, 1% penicillin/streptomycin, and 2 mM l-glutamine.

### Establishment of drug resistance model in vivo.

A murine spontaneous breast tumor model, MMTV-PyMT, was administrated with DDP. Briefly, treatment with DDP (2 mg/kg, i.p., every 2 days) was started when tumors became palpable (around ~100 mm^3^ of median volume). The tumor growth was substantially delayed in treated mice. After this initial period of sensitivity of approximately 30 days, despite continued treatment, the tumors started to grow, indicating the development of acquired resistance. When the tumors reached the volume of 250 mm^3^, tumor growth kinetics of treated mice were similar to those of the untreated control mice. To verify that cells were truly resistant to DDP, both control and resistant tumors were excised and disaggregated, and the tumor cells obtained were treated ex vivo with DDP for 48 hours. Apoptosis in the cells was analyzed using annexin V staining by flow cytometry (CytoFLEX, Beckman Coulter). For in vivo experiments, tumor-bearing mice were administrated with DDP (2 mg/kg), 6-AN (1 mg/kg), GPI (10 mg/kg), or NAC (300 mg/kg) via i.p. injection once every 2 days. In addition, 100 μg SR1 was intratumorally injected into mice once every 2 days. The following experiments were conducted accordingly.

### Establishment of drug-resistant cell lines including MCF-7/DDP, MCF-7/ADR, A549/5-Fu, A549/DDP, HCT116/Oxa, SW1990/Gem, B16/TAX, and 4T-1/DDP by increasing drug concentration.

Methods were based on a previous study (49). Briefly, we took the tumor cells in the logarithmic growth period (confluence 60%–80%), added the drugs with the initial concentration of low concentration (recommended as 1/10–1/5 of IC_50_ of the parental cell line) for 24 hours, discarded the culture medium, washed it twice with PBS, and replaced the medium without drugs. After the cells resumed growth, the cells were treated with low concentration for 24 hours after digestion and subculture. After the cells proliferated to normal morphology, we repeated the above drug shocks with each concentration 6 to 8 times. After the cells grew stably at this concentration, we increased the drug concentration and continued to culture, and the drug was added in increasing concentrations in turn. The drug induction lasted for 6 to 8 months until the cells could grow stably in the concentration of the drug. We detected the IC_50_ of drug-resistant cell lines, then calculated the resistance index (RI) according to the IC_50_ value. RI = IC_50_ of drug-resistant cell lines/IC_50_ of parental cell lines. If RI > 5, it was considered that the drug resistance of drug-resistant cell lines met the requirements of drug-resistant strains. All cells were grown at 37°C in a 5% CO_2_ incubator. Cells were tested for mycoplasma detection and interspecies cross contamination and authenticated by isoenzyme and short-tandem repeat analyses in the Cell Resource Centre of Peking Union Medical College before the study.

### Human samples.

The breast cancer tissues or lung cancer tissues were obtained from patients at the National Cancer Center/Cancer Hospital. Paraffin-embedded tumor tissues of patients with melanoma were obtained from the Department of Pathology, National Cancer Center/Cancer Hospital. The clinical features of the patients are listed in Supplemental Tables 1–4.

### RNA interference and CRISPR/Cas9 construction.

MCF-7/DDP and A549/5-Fu cells were transfected with siRNA interfering target gene expression. All the siRNAs were transfected using Lipofectamine RNAiMAX Transfection Agent (Life Technologies, Thermo Fisher Scientific). siRNA sequences were control siRNA: CGUACGCGGAAUACUUCGA; *G6PD* #*1* siRNA: GCACCTACAAGTGGGTGAA, *G6PD* #*2* siRNA: GCGTTATCCTCACCTTCAA; *GYS1* #*1* siRNA: GCUAUGAGUUCUCCAACAAGG; *GYS1* #*2* siRNA: ACACGGUGCUGCAGACGAAGG; *ME1* #*1* siRNA: CCAGGTTCTTAGAGTAGTA; *ME1* #*2* siRNA: GAACAAACTGTCTGATCAA; *ME2* #*1* siRNA: GAAGAAGCATATACACTTA; *ME2* #*2* siRNA: GCCTTACGATTTCATAGAA; *ME3* #*1* siRNA: GGAACGAGAAGCUCUUCUACC; *ME3* #*2* siRNA: GGCUGUGACAGACAAGUUUGG; *MTHFD2* #*1* siRNA: GGATCAGTATTCCATGTTA; *MTHFD2* #*2* siRNA: GAATGCCCATTGCAATGTT; *AHR* #*1* siRNA: CAGACAGUAGUCUGUUAUAAC; *AHR* #*2* siRNA: CCCAGACAGUAGUCUGUUAUA; *PYGL* #*1* siRNA: GAUUGGAUAUAGAAGAGUUAG; *PYGL* #*2* siRNA: CAAGCUUGGAUUGGAUAUAGA; *GPX* #*1* siRNA: AUUCAGAAUCUCUUCGUUCUU; *GPX* #*2* siRNA: UGGUAUUUUCUGUAAGAUCAG; *GRX* #*1* siRNA: UGUUUGAUGGGCAAUUGACUG; and *GRX* #*2* siRNA: UGAUAUCGACAAAUUCCAGAA. For construction of the stable KO of *AHR* or *PYGL*-MCF-7/DDP and A549/5-Fu cells, the following single-guide RNAs targeting *AHR* or *PYGL* were used: *AHR-SG1*: CACCGTCAAGTCAAATCCTTCCAAG (sense) and AAACCTTGGAAGGATTTGACTTGAC (antisense); *AHR-SG2*: CACCGTTAATAACATCTTGTGGGAA (sense) and AAACTTCCCACAAGATGTTATTAAC (antisense); *PYGL-SG1*: CACCGCCGGCACCTGCACTTCACGC (sense) and AAACGCGTGAAGTGCAGGTGCCGGC (antisense); and *PYGL-SG2*: CACCGTGACGGACCAGGAGAAGCGG (sense) and AAACCCGCTTCTCCTGGTCCGTCAC (antisense).

### Quantitative PCR analysis.

RNA was isolated from cells in triplicate wells in each condition by using TRIzol (Life Technologies, Thermo Fisher Scientific). Generally, 2 μg of RNA for each sample was reversed to cDNA by First-Strand cDNA Synthesis System (Applied Biosystems, Thermo Fisher Scientific). All real-time PCR reactions were performed using the Real-Time PCR System (Applied Biosystems, Thermo Fisher Scientific), and the amplifications were done using SYBR Green PCR Master Mix (Applied Biosystems, Thermo Fisher Scientific). Quantitative PCR primer sequences were *PYGL*-Forward: TGCCCGGCTACATGAATAACA; *PYGL*-Reverse: TGTCATTGGGATAGAGGACCC; *PYGB*-Forward: AGGTGCGGAAGAGCTTCAAC; *PYGB*-Reverse: TCGCGCTCGTAGTAGTGCT; *PYGM*-Forward: GGAACGGATGGACTGGGAC; *PYGM*-Reverse: CAGCGTCTCCAAGAGGTGC; *PPP1R3A*-Forward: TCAACCACTTTTGACTTAGGGAC; *PPP1R3A*-Reverse: ACTTGTAGACCCAAGAAGAGACT; *PPP1R3B*-Forward: TTCGATGACCCGCTAGATATGC; *PPP1R3B*-Reverse: CGGCCTGAAGTCGATTTCTAAA; *PPP1R3C*-Forward: ATCCAGGTTTTAGATCCACGTCC; *PPP1R3C*-Reverse: TGTCGTCGTTGAAATTCATCGT; *PPP1R3D*-Forward: CACCTTCGGCTTTCCAGTACC; *PPP1R3D*-Reverse: GTCTCGGTGGTCGTTGTTGT; *PPP1R3E*-Forward: ATCTCCCAGCCTTAGTCTTTGA; *PPP1R3E*-Reverse: GGTGATCCACATGGTACATGACA; *PPP1R3F*-Forward: AGGTTTCTGACGTTCCGATGA; *PPP1R3F*-Reverse: GGAGGACCTCTGTAAAAGCCA; *PPP1R3G*-Forward: GGCAGTGTTCTCAGTGTT; *PPP1R3G*-Reverse: GTAAGGACCAAGTCTCAAGT; *NOX2*-Forward: ACCGGGTTTATGATATTCCACCT; *NOX2*-Reverse: GATTTCGACAGACTGGCAAGA; *RAC1*-Forward: ATGTCCGTGCAAAGTGGTATC; *RAC1*-Reverse: CTCGGATCGCTTCGTCAAACA; *OCT4*-Forward: CTGGGTTGATCCTCGGACCT; *OCT4*-Reverse: CCATCGGAGTTGCTCTCCA; *SOX2*-Forward: GCCGAGTGGAAACTTTTGTCG; *SOX2*-Reverse: GGCAGCGTGTACTTATCCTTCT; β*CATENIN*-Forward: AAAGCGGCTGTTAGTCACTGG; β*CATENIN*-Reverse: CGAGTCATTGCATACTGTCCAT; *VIMENTIN*-Forward: GACGCCATCAACACCGAGTT; *VIMENTIN*-Reverse: CTTTGTCGTTGGTTAGCTGGT; *SNAIL*-Forward: TCGGAAGCCTAACTACAGCGA; *SNAIL*-Reverse: AGATGAGCATTGGCAGCGAG; β*ACTIN*-Forward: GGCTGTATTCCCCTCCATCG; and β*ACTIN*-Reverse: CCAGTTGGTAACAATGCCATGT.

### ROS detection.

ROS levels were measured using CellROX Green (C10492, Invitrogen, Thermo Fisher Scientific) or MitoSOX Green (M36008, Invitrogen, Thermo Fisher Scientific) flow cytometry assay kits. After treatment, cells were loaded with 5 μM CellROX Green or MitoSOX Green for 30 minutes at 37°C protected from light, then washed in PBS and immediately analyzed by flow cytometry, using 488 nm excitation for the ROS.

### H_2_O_2_ assay.

The peroxide concentrations in cells were detected using the Hydrogen Peroxide Assay Kit (ab102500/K265-200, Abcam). The concentration of peroxide in the samples was calculated by comparing the relative fluorescence units of each sample to the standard curves, which were prepared at the same time.

### Live-cell imaging of HyPerRed fluorescence.

MCF-7 or MCF-7/DDP cells stably expressing HyPerRed (Addgene) were treated with chemo drugs for 24 hours. Cells were imaged on a Nikon AX confocal microscope with the emission spectra of the fluorescence excited at 575 nm. Fluorescence was quantified by ImageJ (NIH).

### NADPH/NADP^+^ assay.

The NADPH/NADP^+^ ratio was determined with NADPH/NADP^+^ Quantification Kit (K347, BioVision).

### GSH/GSSG assay.

The GSH/GSSG ratio was determined with GSH/GSSG Detection Assay Kit (ab138881, Abcam).

### Recombinant AHR protein expression and purification.

Recombinant AHR protein was produced in HEK293T cells (China Center for Type Culture Collection). Briefly, 20 μg Flag-tagged human AHR plasmid was transfected per 15 cm plate of approximately 80% confluent HEK293T cells, using Lipofectamine 3000. At 48 hours after transfection, cells were harvested, lysed on ice for 30 minutes in lysis buffer (50 mM Tris-HCl pH 7.5, 300 mM NaCl, 1 mM EDTA, 1% Triton X-100, 1× Protease/Phosphatase Inhibitor Cocktail), and then sonicated for 3 cycles. The lysate was centrifuged at 10,000*g* at 4°C for 1 hour to remove cell debris. For Flag-AHR protein purification, the supernatant was incubated with anti-Flag M2 beads (MilliporeSigma) at 4°C for 1 hour. The beads were washed 3 times with buffer containing 50 mM Tris-HCl pH 7.5, 500 mM NaCl, 3 mM EDTA, 0.5% NP-40, 10% glycerol, and 0.1 mM DTT. The proteins were eluted with buffer containing 25 mM HEPES (pH 7.4), 150 mM NaCl, and 200 μg/mL 3× Flag peptide (MilliporeSigma). Protein was analyzed by 12% SDS-PAGE with Coomassie blue staining and Western blot.

### Glycogen particulate isolation.

Glycogen particulate was isolated from cells as previously described (39). Briefly, cells were lysed by sonication, and nuclei and cell debris were pelleted by centrifugation at 2,500*g* at 4°C for 5 minutes. Where indicated, the resulting postnuclear supernatant was subjected to sequential centrifugation to prepare plasma membranes (10,000*g*, 4°C, 15 minutes) and to separate glycogen-enriched pellets from the cytosol (100,000*g*, 4°C, 1 hour). Glycogen particulate fractions were resuspended in homogenization buffer using a 23-gauge needle.

### BLI.

The recombinant human AHR protein was purified according to the method described in the former context, while the HSP90 protein (active) was purchased from Abcam. Protein interactions were measured and analyzed by an Octet Red instrument (Pall). The Octet SSA (Super Streptavidin) Biosensors (Pall) were dipped into solution containing HSP90 protein (all solution was kept in 1 μg/mL) and subsequently loaded with different doses of H_2_O_2_-treated AHR protein solution. The protein association and disassociation processes were monitored and analyzed by Octet software (Pall) and processed and graphed with GraphPad software.

### Co-IP and Western blot.

Briefly, indicated plasmids were transfected into HEK293T cells, or tumor cells were treated with chemo drugs for 24 hours or H_2_O_2_ for 30 minutes. Cells were lysed with IP buffer, sonicated, and centrifuged at 10,000*g*, at 4°C for 15 minutes. The supernatants were subjected to IP by incubating them with anti-Flag M2 beads (MilliporeSigma) or anti-Myc or anti-HA Agarose Beads (K.T. Health) for 3 hours or with indicated antibodies overnight at 4°C before incubating with Protein A/G beads (Roche) for 3 hours. The beads were washed 5 times with IP buffer, and the protein complexes were denatured using 2× SDS Loading Buffer, resolved by SDS-PAGE gels, and then transferred to NC membranes. Primary antibodies against indicated genes were used. Peroxidase-conjugated secondary antibody was used, and the antigen-antibody reaction was visualized by enhanced chemiluminescence assay (Thermo Fisher Scientific). Primary antibodies included G6PD (D5D2, Cell Signaling Technology [CST]), β-actin (13E5, CST), PYGL (ab223788, Abcam), p-PYGL (EPR20881-72, Abcam), AHR (D5S6H, CST), STBD1 (1A2G2, Proteintech), AIP (EPR13585, Abcam), HSP90 (D7a, Abcam), dimedone (ABS30, MilliporeSigma), Myc (9B11, CST), Flag (D6W5B, CST), p-Ser/Thr (AF5725, Beyotime), PHKG1 (EPR14812, Abcam), PTG (AB2851218, Thermo Fisher Scientific), PP1c (ab245664, Abcam), and HA (C29F4, CST). Secondary antibodies included HRP-conjugated Affinipure Goat Anti-Mouse IgG (SA00001-1, Proteintech) and HRP-conjugated Affinipure Goat Anti-Rabbit IgG (SA00001-2, Proteintech).

### Single–tumor cell suspension preparation.

Clinical cancer samples or animal tumor tissues were processed immediately after being obtained. Every sample was washed with PBS, cut into small pieces (< 1 mm^3^), transferred into 5 mL DMEM containing collagenase IV (Gibco) (1 mg/mL), and subsequently incubated for 60 minutes on a 37°C shaker. Subsequently, 4 mL PBS was added to dilute the suspension, and then a 70 μm cell mesh (BioFIL) was used to filter the suspension. After centrifugation at 60*g*, at 4°C, for 5 minutes, we collected the cell pellet and resuspended it with cell preservation liquid. Single tumor cells were isolated from Tumor Cell Isolation Kit, human or mouse (Miltenyi Biotec).

### Cysteine sulfenylation labeled by dimedone.

Cysteine sulfenylation (SOH directed) was labeled by 5,5-dimethyl-1,3-cyclohexanedione (dimedone) as previously described (49). Briefly, cells of interest were grown in the appropriate medium to 60% to 90% confluence in 100 mm dishes. The cells were treated with chemo drugs or H_2_O_2_ for indicated times, then switched to medium containing 5 mM dimedone for a total of 30 minutes during this incubation. Following labeling, PBS was used to wash the cells 3 times to remove the excess dimedone. For further biochemical analyses, the cells or cell lysates containing labeled proteins were analyzed by other methods.

### Assessment of AHR cysteine sulfenylation using dimedone MS.

For in-gel trypsin digestion, the gel band of interest was excised from the gel, reduced with 5 mM DTT, and alkylated with 11 mM iodoacetamide. Then the gel band was digested with sequencing-grade modified trypsin at 37°C followed by chymotrypsin at 25°C overnight. The peptides were extracted twice with 1% trifluoroacetic acid in 50% acetonitrile aqueous solution for 30 minutes. The peptide extracts were then centrifuged in a SpeedVac (Thermo Fisher Scientific) to reduce the volume. For LC-MS/MS analysis, the digestion products were separated by 85-minute gradient elution at a flow rate 0.300 μL/min with a Thermo Fisher Scientific Dionex Ultimate 3000 HPLC system, which was directly interfaced with the Thermo Fisher Scientific Orbitrap Exploris 480 mass spectrometer. The analytical column was a homemade fused silica capillary column (75 μm inner diameter, 150 mm length; Upchurch) packed with C-18 resin (100 Å, 2 μm, Dr. Maisch). Mobile phase A consisted of 0.1% formic acid, and mobile phase B consisted of 100% acetonitrile and 0.1% formic acid. The Orbitrap Exploris 480 mass spectrometer was operated in the data-dependent acquisition mode using Xcalibur 3.0 software (Thermo Fisher Scientific), and there was a single full-scan mass spectrum in the Orbitrap (350–1,500 *m/z*, 60,000 resolution) followed by top-speed MS/MS scans in the Orbitrap. The MS/MS spectra from each LC-MS/MS run were searched against the AHR sequence using an in-house Proteome Discoverer (Version PD1.4, Thermo Fisher Scientific). The search criteria were as follows: full tryptic-chymotrypsin specificity was required; 2 missed cleavage sites were allowed; and oxidation (M), deoxidation (C), trioxidation (C), and carbamidomethyl (C) were set as variable modifications. Dimedone on cysteine residues (+138.0681 Da) was set as variable modifications; precursor ion mass tolerance was set at 20 parts per million for all MS acquired in an Orbitrap mass analyzer; and the fragment ion mass tolerance was set at 0.02 Da for all MS/MS spectra acquired. The peptide FDR was calculated using Fixed Value PSM Validator provided by PD. When the *q* value was smaller than 1%, the peptide spectrum match (PSM) was considered correct. FDR was determined based on PSMs when searched against the reverse decoy database. Peptides assigned only to a given protein group were considered unique. The FDR was also set to 0.01 for protein identifications.

### Immunofluorescence staining.

For cells, 4% paraformaldehyde-fixed, treated cells were rinsed with PBS; permeabilized with 0.2% Triton X-100 in PBS for 10 minutes at 4°C; blocked with 5% BSA at room temperature (RT) for 30 minutes; and then incubated with primarily antibodies anti-AHR (1:500; R&D Systems, Bio-Techne, catalog AF6185), anti-STBD1 (1:500; Proteintech, 167018-lg), or anti-dimedone (1:100, Merck Millipore, ABS30) at 4°C for overnight, followed by PBS rinsing and incubating with secondary antibodies for another 1 hour at RT. Secondary antibodies included Alexa Fluor 488 goat anti-mouse IgG(H+L) (A11001, Invitrogen), Alexa Fluor 488 donkey anti-sheep IgG(H+L) (A11015, Invitrogen), Alexa Fluor 594 donkey anti-rabbit IgG(H+L) (A21207, Invitrogen), Alexa Fluor 647 donkey anti-mouse IgG(H+L) (A31571, Invitrogen), and Alexa Fluor 647 goat anti-rabbit IgG(H+L) (A21245, Invitrogen). Finally, after 3 PBS washes, the cells were mounted with VECTASHIELD mounting medium (Vector Laboratories) containing DAPI. All images were collected with a confocal microscope (Nikon, A1R) or super-resolution microscope (General Electric, Delta Vision OMX SR).

### Immunohistochemistry.

Paraffin-embedded samples were sectioned at 3 μm thickness. The tissue sections were deparaffinized and rehydrated and incubated in 3% H_2_O_2_ for 15 minutes, boiled with citrate buffer (pH 6.0) for antigen retrieval, and then blocked with 5% serum followed by incubating overnight at 4°C with primary antibodies including anti-AHR (1:100; GeneTex; catalog GTX22769) and anti–p-PYGL (1:100; Abcam; catalog ab227043). After washing with PBS, tissue sections were incubated with secondary antibodies including Goat Anti-Rabbit IgG H&L (HRP) (ab6721, Abcam) and Goat Anti-Mouse IgG H&L (HRP) (ab205719, Abcam) at RT for 30 minutes, and the immunodetection was performed using DAB (Dako) according to the manufacturer’s instructions. The Pannoramic MIDI microscope and SlideViewer software (both from 3D HISTECH) were used for panoramic scanning of immunohistochemistry.

### Metabolite analysis.

For detection and analysis of R5P, S7P, and E4P, cells were treated with chemo drugs for 24 hours. To investigate the flux of glycogen-derived G6P, cells were cultured in ^13^C-glucose to label the glycogen for 10 days. Cells were pretreated with GPI for 2 hours and then switched to medium with chemo drugs and normal glucose for 1 or 3 hours. For metabolomics analysis, cells cultured in normal glucose were switched to ^13^C-glucose medium containing chemo drugs. The cells were washed twice in saline and lysed in extraction solvent (80% methanol/water) for 30 minutes at –80°C. After centrifugation at 12,000*g*, 10 minutes, at 4°C, supernatant extracts were analyzed by LC-MS as described previously. The LC-MS portion of the platform was based on HPLC (Vanquish Horizon UHPLC system, Thermo Fisher Scientific) and a Q Exactive Mass Spectrometer (Thermo Fisher Scientific). LC used the following 2 analytical methods. (i) The samples were separated on Xbridge amide column (130 Å, 2.1 mm inner diameter, 100 mm length; Waters). The mobile phase A was 20 mM ammonium acetate and 15 mM ammonium hydroxide in water with 3% acetonitrile, pH 9.0, and mobile phase B is acetonitrile. The linear gradient was as follows: 0 minute, 85% B; 1.5 minutes, 85% B, 5.5 minutes, 30% B; 8 minutes, 30% B, 10 minutes, 85% B, and 12 minutes, 85% B. The flow rate was 0.2 mL/min. (ii) Metabolites were separated on a 150 mm × 2.1 mm, 2.7 mm Acquity UPLC BEH C18 Column (Waters) with a gradient of solvent A (5 mM N,N-Dimethyloctylamine, H_2_O, pH 5.5) and solvent B (5 mM N,N-Dimethyloctylamine, 90% methanol/H_2_O, pH 5.5). The gradient was 0 minute, 10% B; 1.5 minutes, 10% B; 5.5 minutes, 100% B; 8 minutes, 100% B; 10 minutes, 10% B; 15 minutes, 10% B. Flow rate was 0.3 mL/min. Sample volumes of 5 mL were injected for LC-MS analysis. Data were quantified by integrating the area underneath the curve of each compound using the Xcalibur Qual browser.

### MS analysis.

To identify the binding protein of glycogen, chemo-treated cells were immunoprecipitated by anti-STBD1 and separated using SDS-PAGE, followed by colloidal Coomassie staining. Binding protein bands were sliced into 2 mm sections and then destained, dried, and incubated with trypsin overnight at 37°C. Finally, the peptide extracts were processed for nano-UPLC separation using a nano-Acquity system. MS raw data files were converted into MGF files for identification and relative quantitation using Proteome Discoverer.

### De novo modeling.

The protein structures of AHR and PTG were predicted by the I-TASSER server (https://zhanggroup.org/I-TASSER/), which is an online resource for automated protein structure prediction and structure-based function annotation. It is a hierarchical template-based method.

### Protein and protein docking.

HDOCK (http://hdock.phys.hust.edu.cn) was used for docking PTG with protein AHR. The HDOCK server predicts the binding complexes between 2 molecules, like proteins and proteins, by using a hybrid docking strategy. In the docking process, AHR was selected as receptor and PTG as ligand. Molecular graphics were generated by PyMOL.

### Molecular dynamics simulation.

The AHR 300C(SH) in the AHR-PTG complex was modified into SOH to obtain the AHR(modified)-PTG complex. The structure of AHR(SOH)-PTG was optimized by molecular dynamics (MD) simulation. Each structure of protein was neutralized by adding sodium/chlorine counter ions and solvated in a cuboid box of TIP3P water molecules with solvent layers 10 Å between the box edges and solute surface. All MD simulations were performed using AMBER161. The AMBER FF14SB force field was applied, and the SHAKE algorithm was used to restrict all covalent bonds involving hydrogen atoms with a time step of 2 fs. The particle mesh Ewald method was used to treat long-range electrostatic interactions. For each solvated system, 2 steps of minimization were performed before the heating step. The first 4,000 cycles of minimization were performed with all heavy atoms restrained with 50 kcal/(mol·Å^2^), whereas solvent molecules and hydrogen atoms were free to move. Then, unrestrained minimization was carried out involving 2,000 cycles of steepest descent minimization and 2,000 cycles of conjugated gradient minimization. Afterward, the whole system was first heated from 0 K to 300 K in 100 ps using Langevin dynamics at a constant volume and then equilibrated for 150 ps at a constant pressure of 1 atm. Periodic boundary dynamics simulations were carried out for the whole system with an NPT (constant composition, pressure, and temperature) ensemble at a constant pressure of 1 atm and 300 K in the production step. In production phase, 100 ns simulation was carried out.

### Bioinformatics analysis.

R language was used for performing statistical analysis and graphical work. For survival analysis, Kaplan-Meier survival curves and the log-rank test were used to evaluate the outcomes of patients in TCGA or GSE cohort with different AHR, PYGL, or G6PD expression according to R package survival.

### Statistics.

All experiments were performed at least 3 times. Results are expressed as mean ± SD as indicated and analyzed by 2-tailed Student’s *t* test or 1-way ANOVA followed by Bonferroni’s test. Survival rate was analyzed by log-rank test. *P* value less than 0.05 was considered statistically significant. The analysis was conducted using GraphPad Prism 8.0.

### Study approval.

All studies involving mice were approved by the Institutional Animal Care and Use Committee (IACUC) of the CAMS (ACUC-A02-2022-095). The maximum tumor size allowed by the IACUC was 20 mm, and animal experiments exceeding this limit should be approved by the IACUC as a special case. The breast cancer tissues or lung cancer tissues were obtained from patients at the National Cancer Center/Cancer Hospital. All patients involved in this study gave written informed consent. Ethical permission was granted by the Clinical Trial Ethics Committee of National Cancer Center/Cancer Hospital (22/384-3586).

### Data availability.

Data are available in the Supporting Data Values XLS file. See complete unedited blots in the supplemental material. All data needed to evaluate the conclusions of this study are available in the paper.

## Author contributions

BH conceived the project. NZ, JC, ZL, CZ, YZ, DW, LZ, ZW, NS, XW, HZ, KT, JM, and JL performed the experiments. NZ, JC, HZ, KT, JM, and JL developed methodology. NZ, JC, JL, and BH analyzed the data. BH and NZ wrote the manuscript.

## Supplementary Material

Supplemental data

Supporting data values

## Version 1 12/15/2023

Electronic publication
