# Supplementary material for: Aryl hydrocarbon receptor sulfenylation promotes glycogenolysis and rescues cancer chemoresistance
Source: J Clin Invest. 2023 Dec 15;133(24):e170753. doi: 10.1172/JCI170753 (PMC10721154; doi:10.1172/JCI170753)
Supplement: Supplemental data [file jci-133-170753-s008.pdf]

Fig. S1 A-M

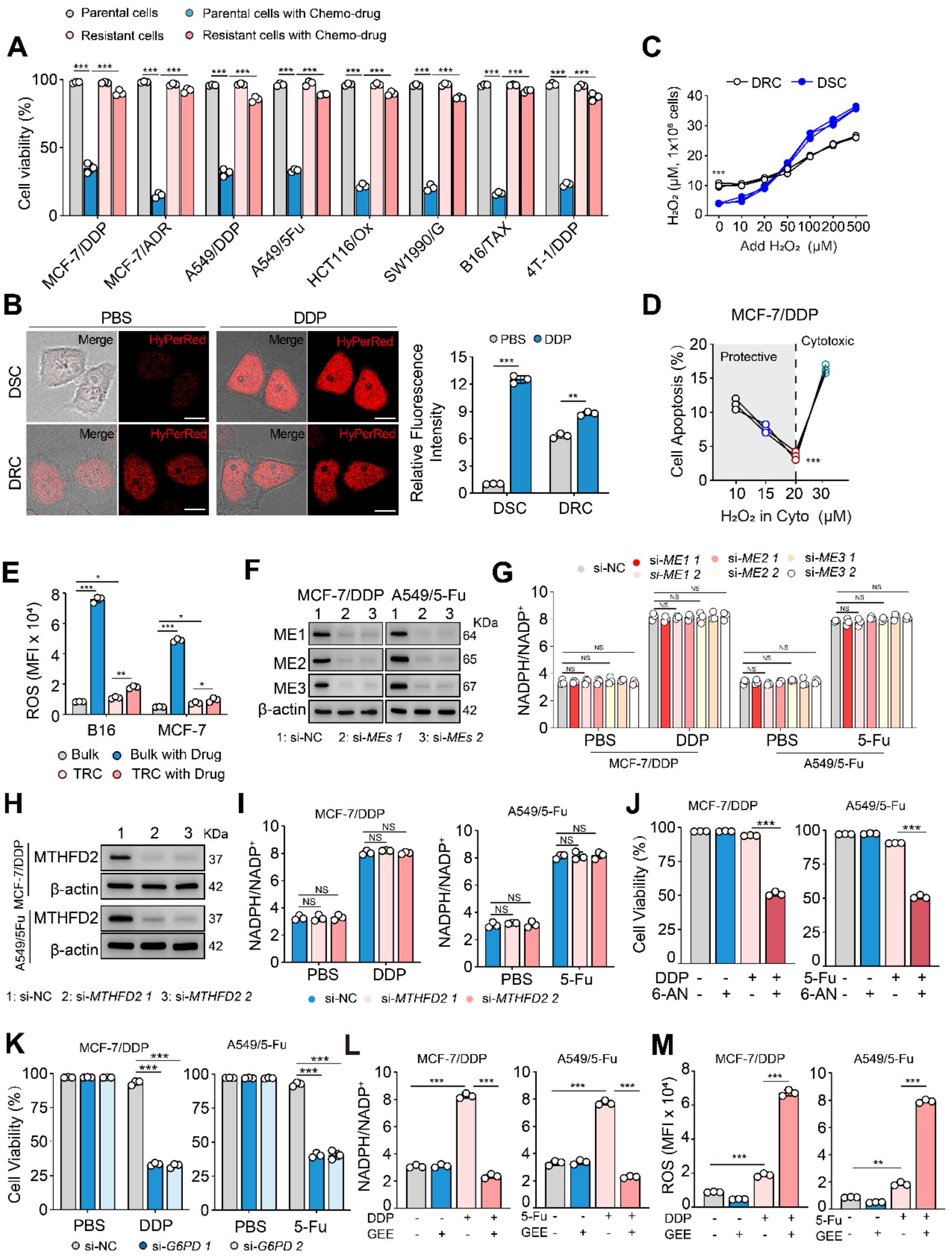

Fig. S1 A-M: DRCs use higher ROS to produce NADPH during treatment. Related to Fig. 1. (**A**) DRCs and DSCs were treated with their corresponding chemo-drugs for 72 hr. Cell viability was analyzed. (**B**) HyPerRed-expressing DRCs (MCF-7/DDP) and DSCs (MCF-7) treated with DDP for 24 hr. MFI was analyzed. Scale bar, 10 μm. (**C**) Different doses of H_2O_2 were treated with DRCs and DSCs for 4hr. Cytoplasmic H_2O_2 concentration was analyzed. (**D**) H_2O_2 pre-treated with DRCs for 4hr were treated with DDP (40 μM) for 48hr. Cell apoptosis was analyzed. (**E**) Bulk or

TRC from B16 and MCF-7 were treated with drugs for 24hr. ROS level was analyzed. (**F**) The expression of MEs in DRCs transduced with si-NC or si-*ME*s was analyzed. (**G**) DRCs transduced with si-NC or si-*ME*s were treated with DDP (20 μM) or 5-Fu (100 μM) for 24 hr. NADPH/ NADP+ was analyzed. (**H** and **I**) The expression of MTHFD2 in DRCs transduced with si-NC or si-*MTHFD2* was analyzed by Western Blot, and were treated with drugs for 24 hr. NADPH/ NADP+ was analyzed. (**J**) DRCs were treated with drugs alone or in combination with 6-AN (50 μM) for 48 hr. The cell viability was analyzed. (**K**) DRCs transduced with si-NC or si-*G6PD* were treated with drugs for 48 hr. Cell viability was analyzed. (**L** and **M**) DRCs were treated with Glutathione reduced ethyl ester (GEE, 5 mM) for 24 hr prior to treatment with drugs. NADPH/ NADP+ (**L**) and ROS levels (**M**) were analyzed. **A**-**M**, n=3. All error bars are mean ± SD, p values were calculated by one-way ANOVA followed by Bonferroni's test (**A**, **D**, **E**, **G**, **I-M**), two-tailed unpaired Student' s t test (**B**, **C**), *p < 0.05, **p < 0.01, ***p < 0.001, NS, not significant (p > 0.05).

Fig. S1 N-W

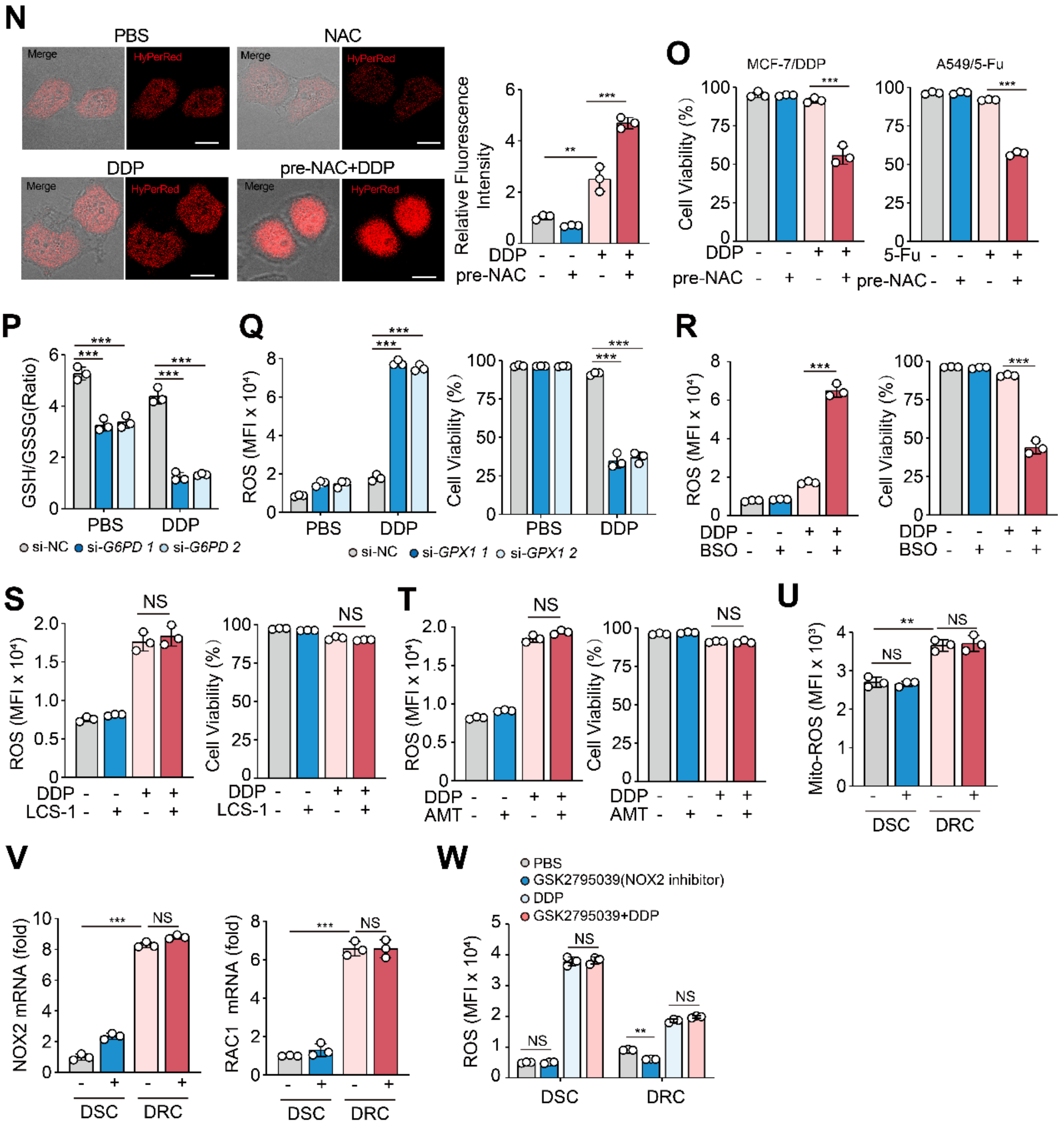

Fig. S1 N-W: DRCs use higher ROS to produce NADPH during treatment. Related to Fig. 1. (**N**) HyPerRed-expressing DRCs pre-treated with NAC (5 mM) for 12hr were treated with DDP for 24 hr. MFI was analyzed. Scale bar, 10 μm. (**O**) DRCs pre-treated with NAC (5 mM) for 12 hr were treated with drugs for 48hr. The cell viability was analyzed. (**P**) DRCs transduced with si-NC or si-*G6PD* was treated with DDP for 24 hr. GSH/GSSG was analyzed. (**Q**) DRCs transduced with si-NC or si-*GPX1* was treated with DDP for indicate times. ROS level (24 hr) and cell viability (48 hr) were analyzed. (**R**) DRCs was treated with DDP alone or in combination with GSH synthesis inhibitor (BSO, 50 μM) for indicate times. ROS level and cell viability were analyzed. (**S**) DRCs was treated with DDP alone or in combination with SOD1 inhibitor (LCS-1, 5 μM) for indicate times. ROS level and cell viability were analyzed. (**T**) DRCs was treated with DDP alone or in combination with CAT inhibitor (AMT, 50 μM) for indicate times. ROS level and cell viability were analyzed. (**U**) DSCs and DRCs were treated with DDP

for 24 hr. Mito-ROS level was analyzed. (**V**) DSCs and DRCs were treated with DDP for 24 hr. The expression of NOX2 and RAC1 was analyzed by Real-time PCR. (**W**) DSCs and DRCs pre-treated with NOX2 inhibitor (GSK2795039, 20 μM) for 12hr were treated with DDP for 24hr. ROS level was analyzed. **N-W**, n=3. All error bars are mean ± SD, p values were calculated by one-way ANOVA followed by Bonferroni's test (**N-W**), *p < 0.05, **p < 0.01, ***p < 0.001, NS, not significant (p > 0.05).

Fig. S2

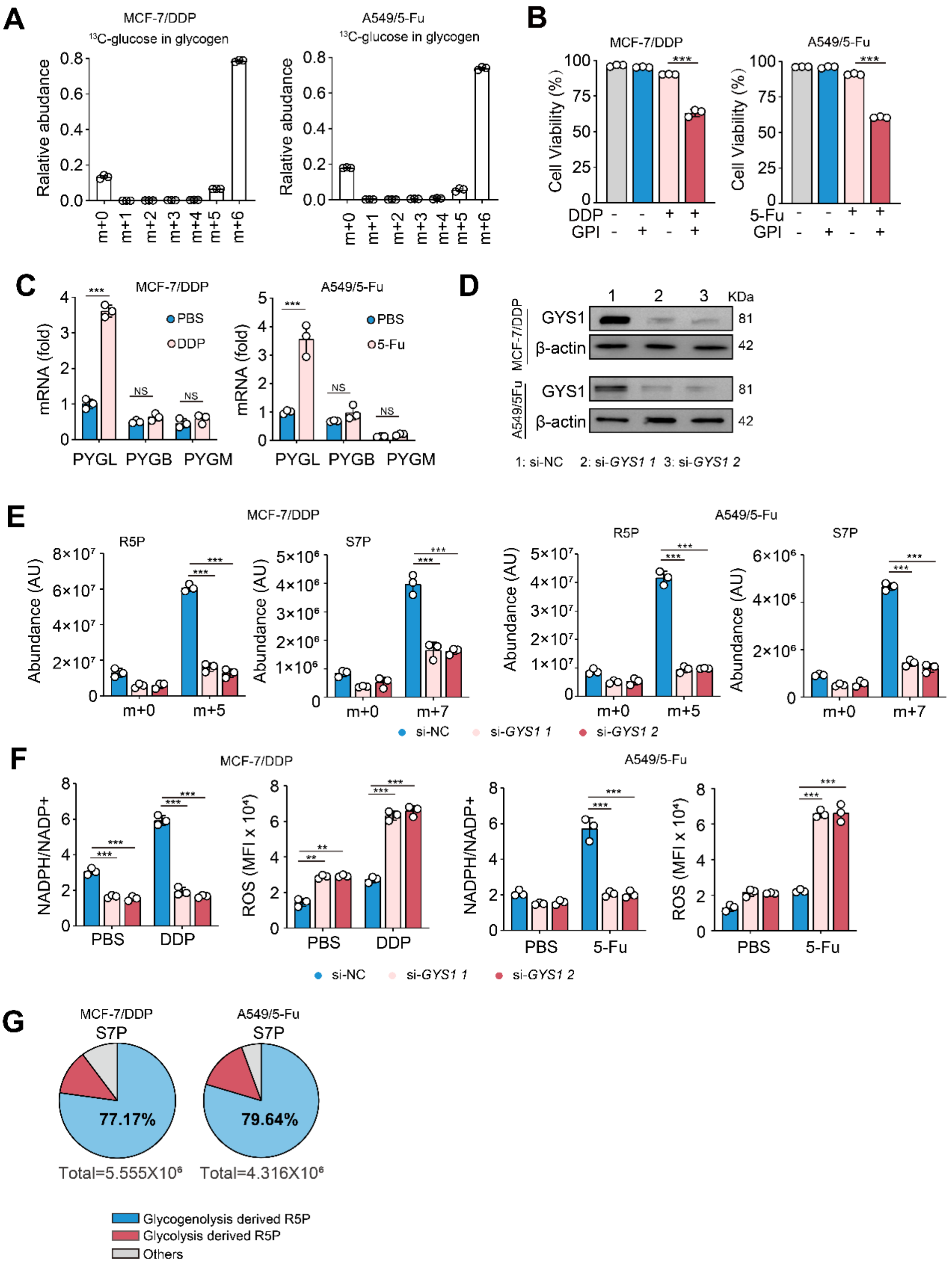

Fig. S2: Glycogenolysis drives PPP in DRCs in response to drug molecules. Related to Fig. 2. (**A**) DRCs were cultured in ^{13}C-glucose for 10 days, followed by the treatment with hydrochloric acid, leading to the degradation of polymer glycogen into monomer glucose. The released ^{13}C-labeled glucose was determined by LC-MS/MS. (**B**) DRCs were treated with drugs alone or in combination with GPI (50 μM) for 48 hr. The cell

viability was analyzed. (**C**) DRCs were treated with drugs for 24 hr, PYGL, PYGB or PYGM expression was determined by Real-time PCR. (**D**) The expression of GYS1 in DRCs transduced with si-NC or si-*GYS1* was analyzed by Western Blot. (**E**) DRCs transduced with si-NC or si-*GYS1* cultured in ^{13}C-glucose were switched to ^{12}C-glucose for 4 hr drug-treatment, and ^{13}C-labeled R5P or S7P was detected by LC-MS/MS. (**F**) DRCs transduced with si-NC or si-*GYS1* were treated with DDP or 5-Fu for 24 hr. The ratio of NADPH/ NADP+ and ROS levels were analyzed. (**G**) DRCs cultured in ^{12}C-glucose pre-treated with GPI for 2 hr were switched to ^{13}C-glucose for 4hr drug-treatment, and ^{13}C-labeled S7P were detected by LC-MS/MS. **A**-**G**, n=3.All error bars are mean ± SD, p values were calculated by one-way ANOVA followed by Bonferroni's test (**B**, **E**, **F**), two-tailed unpaired Student' s t test (**C**), *$p < 0.05$, **$p < 0.01$, ***$p < 0.001$, NS, not significant ($p > 0.05$).

Fig. S3 A-F

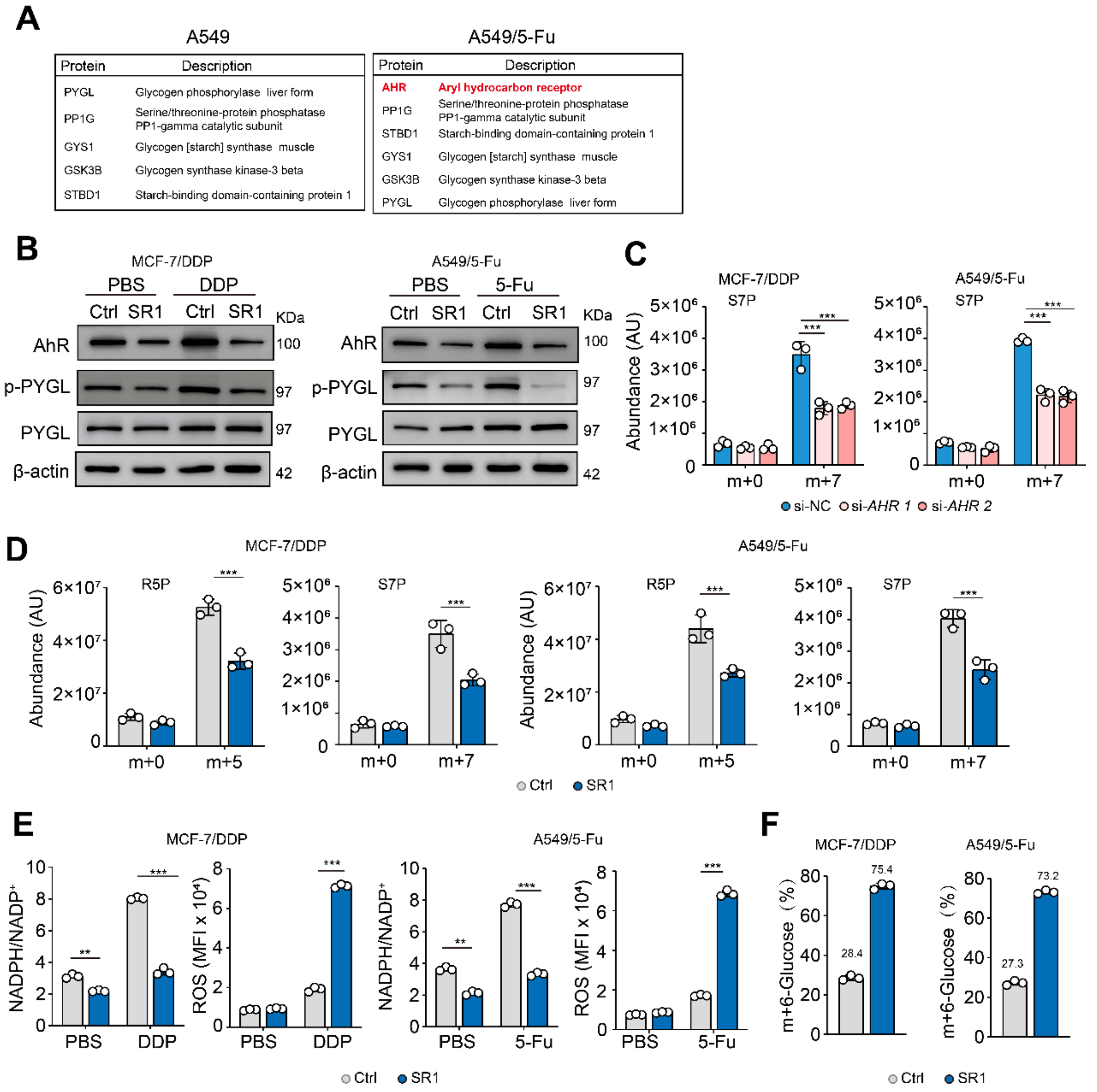

Fig. S3 A-F: DRCs use AHR to promote glycogenolysis. Related to Fig. 3. (**A**) A549 or A549/5-Fu cells were treated with 5-Fu for 24 hr. Cell lysates were immunoprecipitated with anti-STBD1 for mass spectrometry. Identified proteins were listed. (**B**) DRCs were pre-treated with SR1 (10 μM) for 12 hr, and treated with DDP for 24 hr, Phospho-PYGL or PYGL were analyzed by Western Blot. (**C**) DRCs cultured in ^{13}C-glucose transduced with si-NC or si-*AHR* were switched to ^{12}C-glucose for 4 hr drug-treatment, and ^{13}C-labeled S7P was detected by LC-MS/MS. (**D**) DRCs cultured in ^{13}C-glucose were pre-treated with SR1 (10 μM) for 12 hr, and switched to ^{12}C-glucose for 4 hr drug-treatment, and ^{13}C-labeled R5P or S7P was detected by LC-MS/MS. (**E**) DRCs pre-treated with SR1 for 12 hr were treated with DDP or 5-Fu for 24 hr. NADPH/NADP^{+} and ROS levels were analyzed. (**F**) DRCs cultured in ^{13}C-glucose medium for 10 days pre-treated with SR1 for 24 hr were treated with DDP or 5-Fu for 8 hr, followed by the treatment with hydrochloric acid, leading to the degradation of polymer glycogen into monomer glucose. The released ^{13}C-labeled

glucose was determined by LC-MS. All error bars are mean ± SD, p values were calculated by one-way ANOVA followed by Bonferroni's test (**C**), two-tailed unpaired Student's t test (**D**-**F**), n=3, $**p < 0.01$, $***p < 0.001$.

Fig. S3 G-O

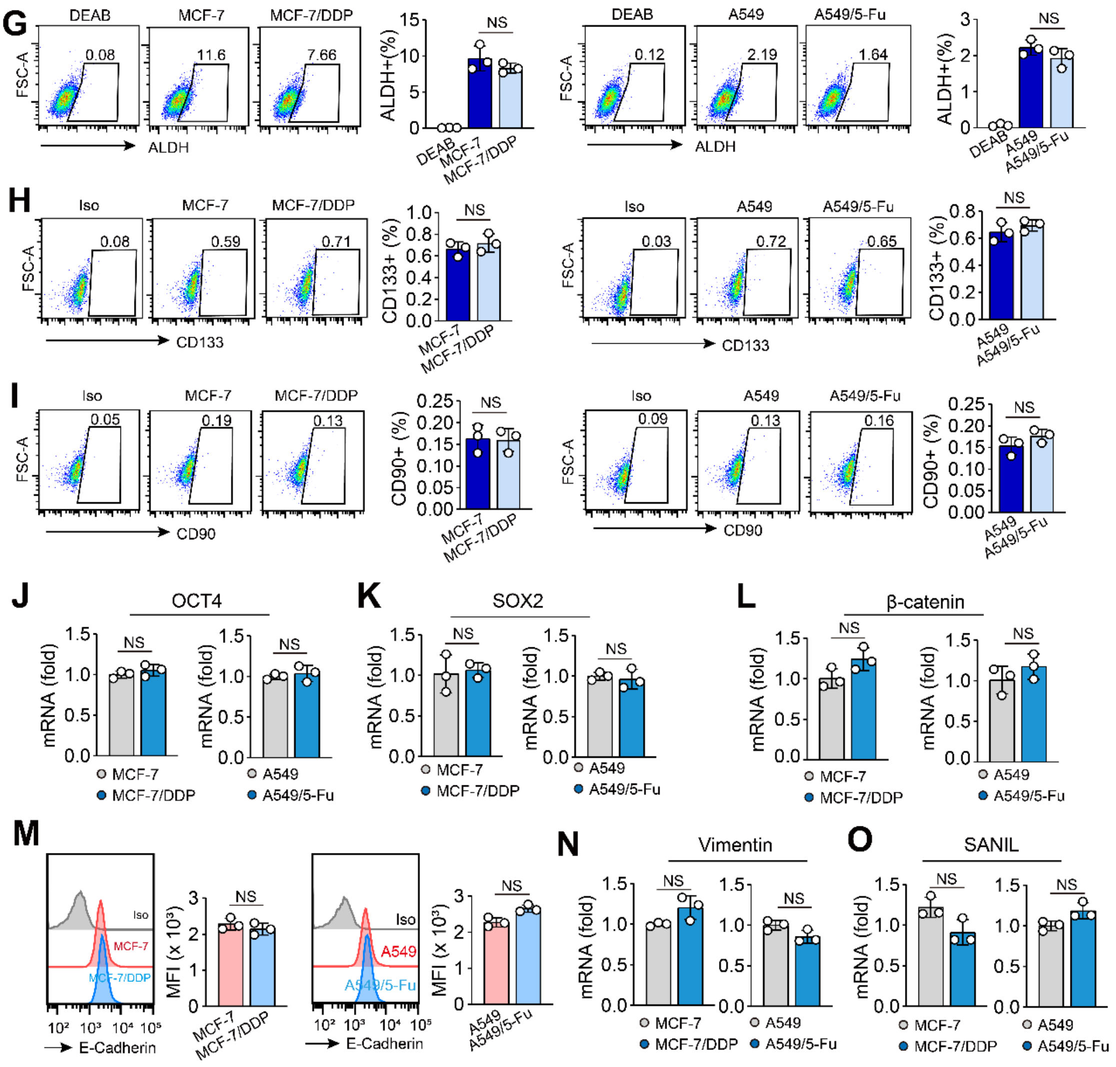

Fig. S3 G-O: DRCs use AHR to promote glycogenolysis. Related to Fig. 3. (**G-I**) The expression of ALDH (**G**), CD133(**H**) and CD90 (**I**) in DSCs (MCF-7, A549) and DRCs (MCF-7/DDP, A549/5-Fu) were determined by flow cytometry. (**J-L**) The expression of OCT4 (**J**), SOX2 (K) and β-catenin (**L**) in DSCs and DRCs were determined by Real-time PCR. (**M**) The expression of E-Cadherin in DSCs and DRCs were determined by flow cytometry. (**N** and **O**) The expression of Vimentin (**N**) and SNAIL (**O**) in DSCs and DRCs were determined by Real-time PCR. **G-O**, n=3. All error bars are mean ± SD, p values were calculated by two-tailed unpaired Student's t test, NS, not significant ($p > 0.05$).

Fig. S4

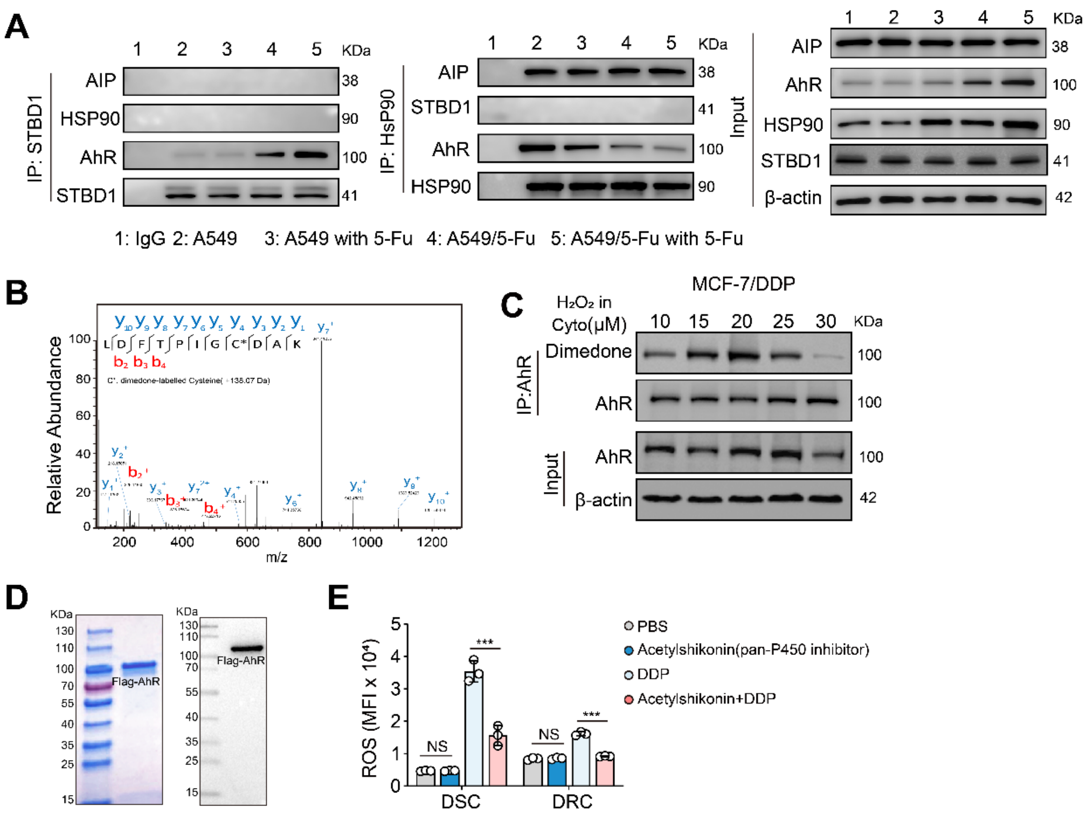

Fig. S4: Cysteine sulfenylation licenses AHR to bind to glycogen particles. Related to Fig. 4. (**A**) Immunoblot of immunoprecipitations of STBD1 or HSP90 in lysates from A549 or A549/5-Fu cells treated with 5-Fu for 24 hr. (**B**) LC-MS/MS analysis of dimedone-labeled AHR in A549/5-Fu cells treated with 5-Fu for 24 hr. Analysis of the y-ions indicates the formation of dimedone adduct (+138.07Da). (**C**) Different dose of H_2O_2 (0, 50, 100, 200, 500 μM) were treated with DRCs, the AHR sulfenylation was analyzed by Western blot. (**D**) Purified Flag-AHR protein was detected by Coomassie Brilliant Blue staining and western blotting. (**E**) DSC(MCF-7) and DRC(MCF-7/DDP) pre-treated with a pan-P450 inhibitor (Acetylshikonin, 10 μM) for 12hr were treated with DDP for 24hr, the ROS level was analyzed. All error bars are mean ± SD, P values were calculated by one-way ANOVA followed by Bonferroni's test (**E**), n=3, $*p < 0.05$, $**p < 0.01$, $***p < 0.001$, NS, not significant ($p > 0.05$).

Fig. S5 A-J

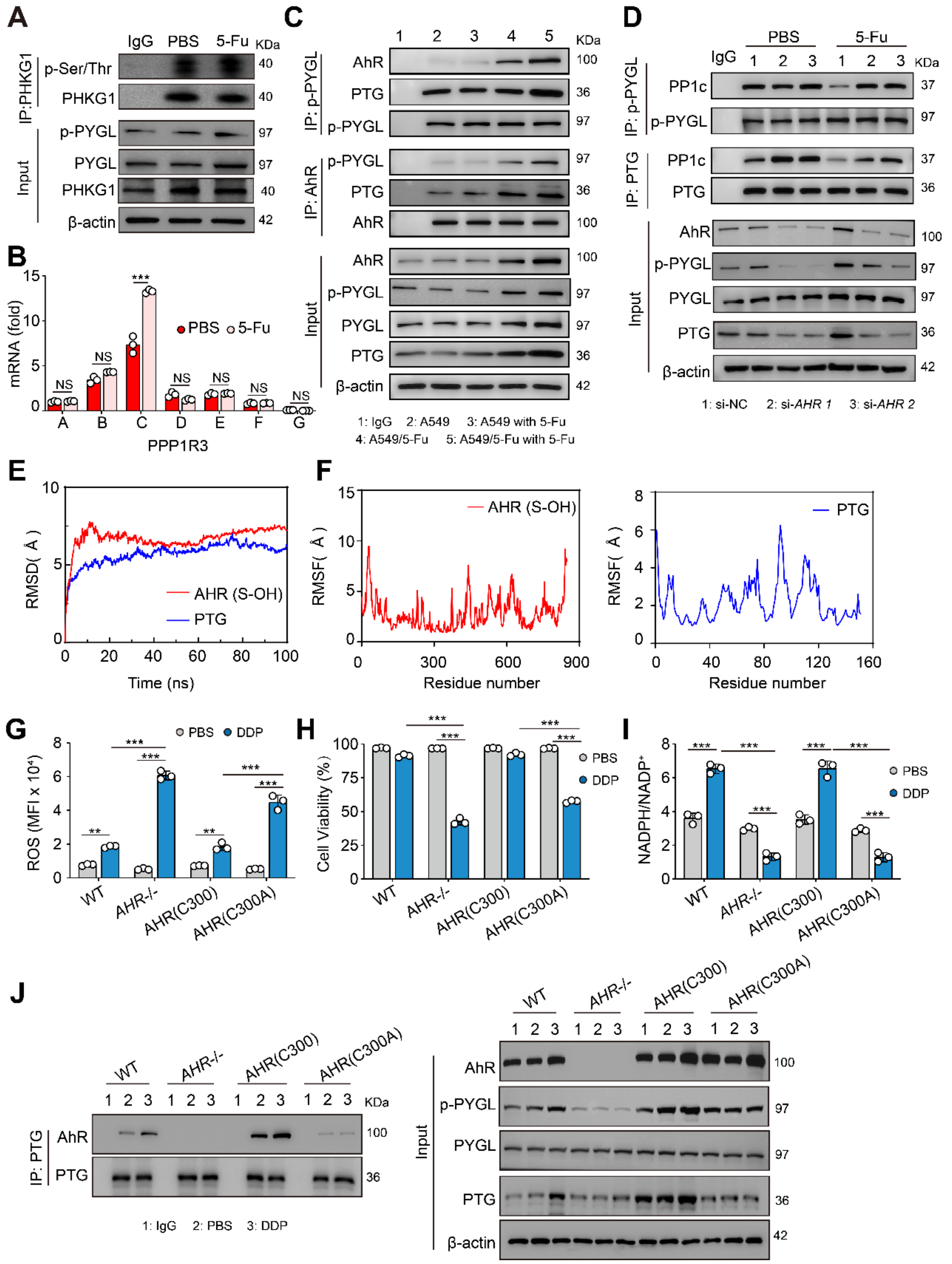

Fig. S5 A-J: Sulfenylated AHR promotes GP activity by competitively binding PTG. Related to Fig. 5. (**A**) A549/5-Fu cells were treated with 5-Fu for 24 hr, Phospho-PHKG1, Phospho-PYGL, PYGL or PHKG1 were analyzed by Western Blot. (**B**) A549/5-Fu cells were treated with 5-Fu for 24 hr, PPP1R3A-G expression was determined by Real-time PCR. (**C**) Immunoblot of immunoprecipitations of p-PYGL

or AHR in lysates from A549 or A549/5-Fu cells treated with 5-Fu for 24 hr. (**D**) Immunoblot of immunoprecipitations of p-PYGL or PTG in lysates from A549/5-Fu cells transfected with si-NC or si-AHR treated with DDP for 24 hr. (**E** and **F**) The tendency of the root mean-square deviation (RMSD) plot for AHR-(SOH)-PTG (**E**), and the root mean-square fluctuation (RMSF) plot for AHR-(SOH) and PTG (**F**). (**G**) WT DRC, *AHR*-/- DRC, *AHR*(C300) DRC and *AHR*(C300A) DRC were treated with DDP for 24 hr, the ROS level was analyzed. (**H**) WT DRC, *AHR*-/- DRC, *AHR* (C300) DRC and AHR(C300A) DRC were treated with DDP for 48 hr, the cell viability was analyzed. (**I**) WT DRC, *AHR*-/- DRC, *AHR*(C300) DRC and *AHR*(C300A) DRC were treated with DDP for 24 hr, the ratio of NADPH/NADP+ were analyzed. (**J**) Immunoblot of immunoprecipitations of PTG in lysates from WT DRC, *AHR*-/- DRC, *AHR*(C300) DRC and *AHR*(C300A) DRC were treated with DDP for 24 hr. All error bars are mean ± SD, p values were calculated by one-way ANOVA followed by Bonferroni's test (**G**-**I**), two-tailed unpaired Student' s t test (**B**), n=3, *$p < 0.05$, **$p < 0.01$, ***$p < 0.001$, NS, not significant ($p > 0.05$).

Fig. S5 K-O

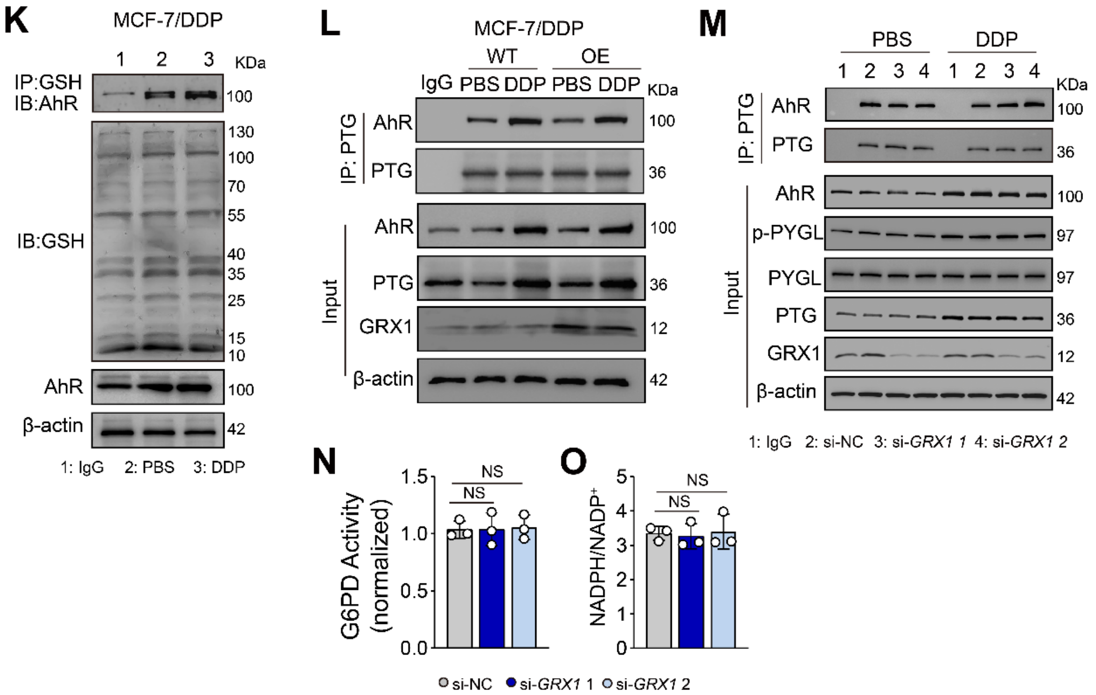

Fig. S5 K-O: Sulfenylated AHR promotes GP activity by competitively binding PTG. Related to Fig. 5. (**K**) Co-IP of S-glutathionylation of AHR in DRC (MCF-7/DDP) (IP, GSH; IB, AHR). (**L**) Immunoblot of immunoprecipitations of PTG in lysates from DRCs or GRX1-over expressing DRCs treated with DDP for 24 hr. (**M**) Immunoblot of immunoprecipitations of p-PYGL or PTG in lysates from DRC (MCF-7/DDP) transfected with si-NC or si-GRX1 treated with DDP for 24 hr. (**N** and **O**) The G6PD activity (N) and the ratio of NADPH/NADP+ (O) in DRCs transfected with si-NC or si-GRX1 was analyzed (n=3). All error bars are mean ± SD, p values were calculated by one-way ANOVA followed by Bonferroni's test (**N, O**), n=3, NS, not significant ($p > 0.05$).

Fig. S6

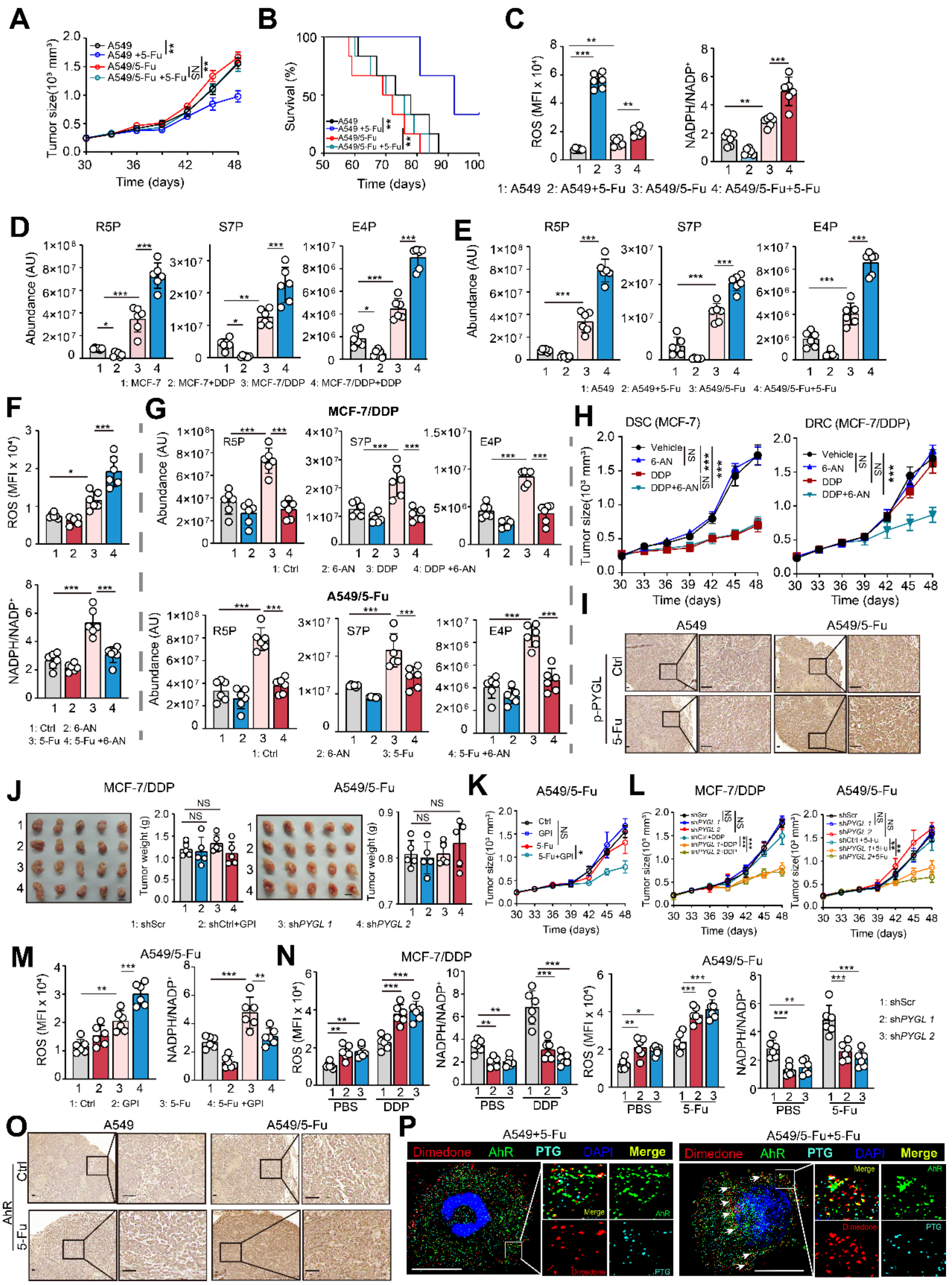

Fig. S6 A-P: Sulfenylated AHR-regulated glycogenolysis promotes drug resistance in vivo. Related to Fig. 6. (**A** and **B**) Tumor-bearing mice were administrated with 5-Fu. Tumor growth (**A**) and mouse survival (**B**) were monitored. (**C**-**E**) Tumor-bearing mice were administrated with drugs for 5 times. ROS levels and NADPH/NADP$^+$ in isolated tumor cells were analyzed (**C**), and R5P, S7P or E4P levels were determined

by LC-MS/MS (**D** and **E**). (**F** and **G**) Tumor-bearing mice were administrated with drugs and 6-AN for 3 times. ROS levels and NADPH/$NADP^+$ were analyzed (**F**), and the R5P, S7P or E4P levels were determined (**G**). (**H**) Tumor-bearing mice were administrated with DDP and 6-AN for 7 times. The tumor growth was monitored. (**I**) Immunohistochemical staining of p-PYGL from the sections of tumor tissues. Scale bars, 50 μm. (**J**) sh-Scr, sh-*PYGL* 1 or sh-*PYGL* 2 DRCs were inoculated into NSG mice. Tumor size was presented photographically (left) or weighted (right). Scale bars, 1 cm. (**K**) Tumor-bearing mice were administrated with 5-Fu and GPI for 5 times. The tumor growth was monitored. (**L**) shScr, sh*PYGL* 1 or sh*PYGL* 2 DRCs inoculated into NSG mice were administrated with drugs for 5 times. The tumor growth of mice was monitored. (**M**) Tumor-bearing mice were administrated with 5-Fu and GPI for 3 times. ROS levels and NADPH/$NADP^+$ were analyzed. (**N**) shScr, sh*PYGL* 1 or sh*PYGL* 2 DRCs inoculated into NSG mice were administrated with drugs for 3 times. ROS levels and NADPH/ NADP+ were analyzed. (**O**) Immunohistochemical staining of AHR from the sections of tumor tissues. Scale bars, 50 μm. (**P**) Isolated tumor cells were labeled with Dimedone. The location of AHR (green) and STBD1 (cyan) was observed under Super-Resolution Microscope. Scale bars, 10 μm. **A**-**G**, **I**, **K**-**P**, n=6 mice; **H**, **J**, n=5 mice. All error bars are mean ± SD, p values were calculated by one-way ANOVA followed by Bonferroni's test (**A**, **C**-**H**, **J**-**N**), and log-rank test (**B**);*p < 0.05, **p < 0.01, ***p < 0.001; NS, not significant (p > 0.05).

Fig. S7

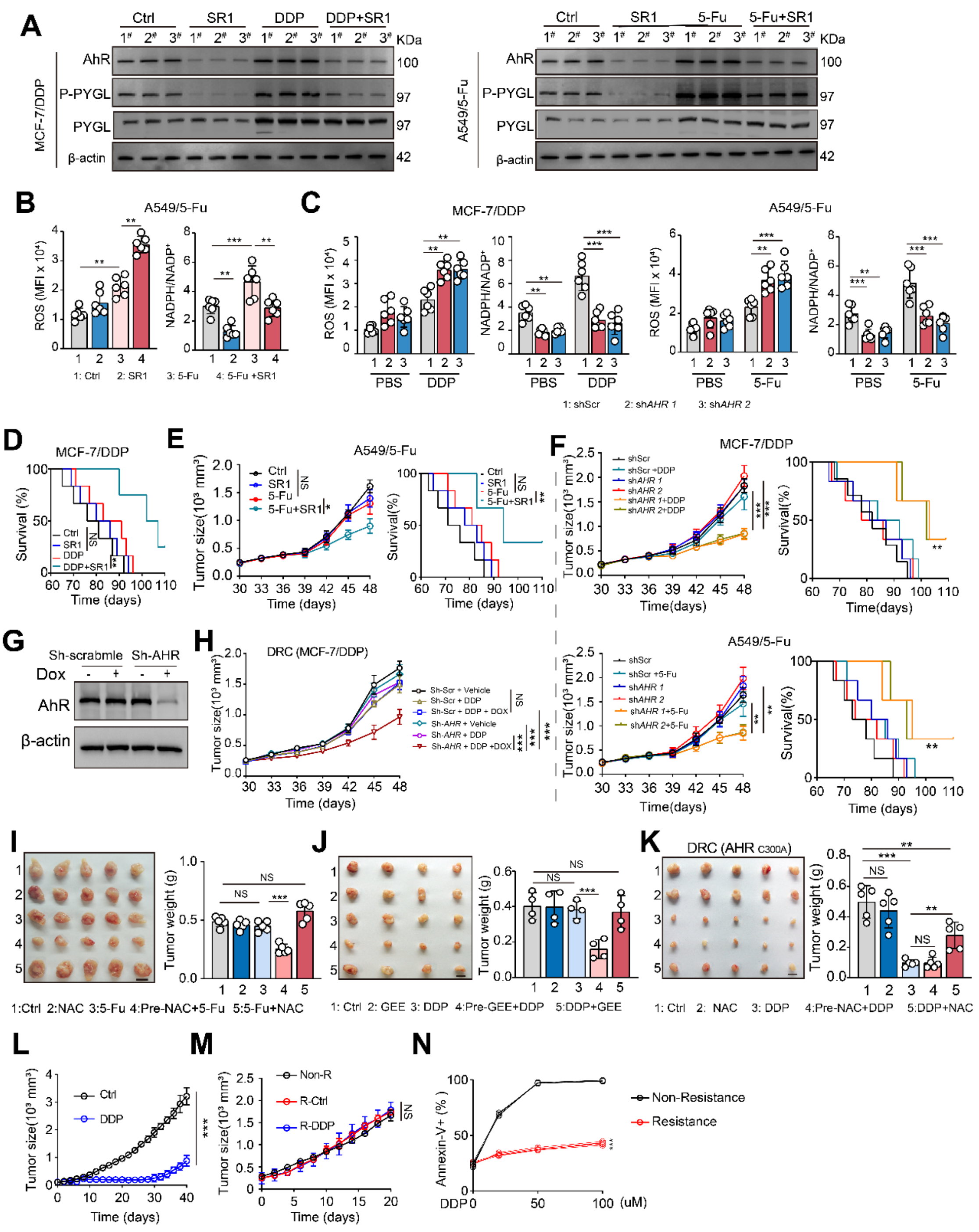

Fig. S7: Sulfenylated AHR-regulated glycogenolysis promotes drug resistance in vivo. Related to Fig. 6. (**A** and **B**) Tumor-bearing mice were administrated with drugs or SR1 for 3 times. p-PYGL and PYGL expression were determined (**A**), and ROS levels or NADPH/NADP$^+$(**B**) in isolated tumor cells were analyzed. (**C**) shScr, sh*AHR* 1 or sh*AHR* 2 were inoculated into NSG mice administrated with drugs for 3 times. ROS levels and NADPH/ NADP+ in isolated tumor cells were analyzed. (**D** and **E**) Tumor-bearing mice were administrated with drugs or SR1. The tumor growth and the survival of mice were monitored. (**F**) shScr, sh-*AHR* 1 or sh*AHR* 2 DRCs were

inoculated into NSG mice administrated with drugs for 5 times. Tumor growth and mouse survival were monitored. (**G**) Immunoblot of AHR in inducible-Sh-Scr- or Sh-AHR- DRCs treated with Dox (1μg/ml) for 48hr. (**H**) Dox inducible-Sh-Scr or Sh-AHR-DRCs were inoculated into NSG mice. Tumor-bearing mice were administrated with drugs and DOX (50 μg/ mouse) for 10 times. The tumor growth was monitored. (**I**-**K**) Tumor-bearing mice were administrated with drugs and NAC or GEE for 5 times, or pre-treated with NAC for 3 days. Tumor size was presented photographically (left) or weighted (right). Scale bars, 1 cm. (**L**) MMTV-PyMT mice were administrated with DDP. The tumor growth was monitored. (**M**) Tumor growth kinetics of the three groups of mice (Non-R/Control, R-Ctrl/Resistance and R-DDP/Resistance with DDP). (**N**) Evaluation of the sensitivity of tumor cells from untreated control group and induced resistance group. **A**, **N**, n=3 mice; **B-F,** n=6 mice; **H, I, K, L, M,** n=5 mice; **J**, n=4 mice. All error bars are mean ± SD, p values were calculated by one-way ANOVA followed by Bonferroni's test (**B, C, E, F, H-K(right)**), two-tailed unpaired Student' s t test (**L-N**), and log-rank test (D, E(right) and F(right));*$p < 0.05$, **$p < 0.01$, ***$p < 0.001$; NS, not significant ($p > 0.05$).

Fig. S8

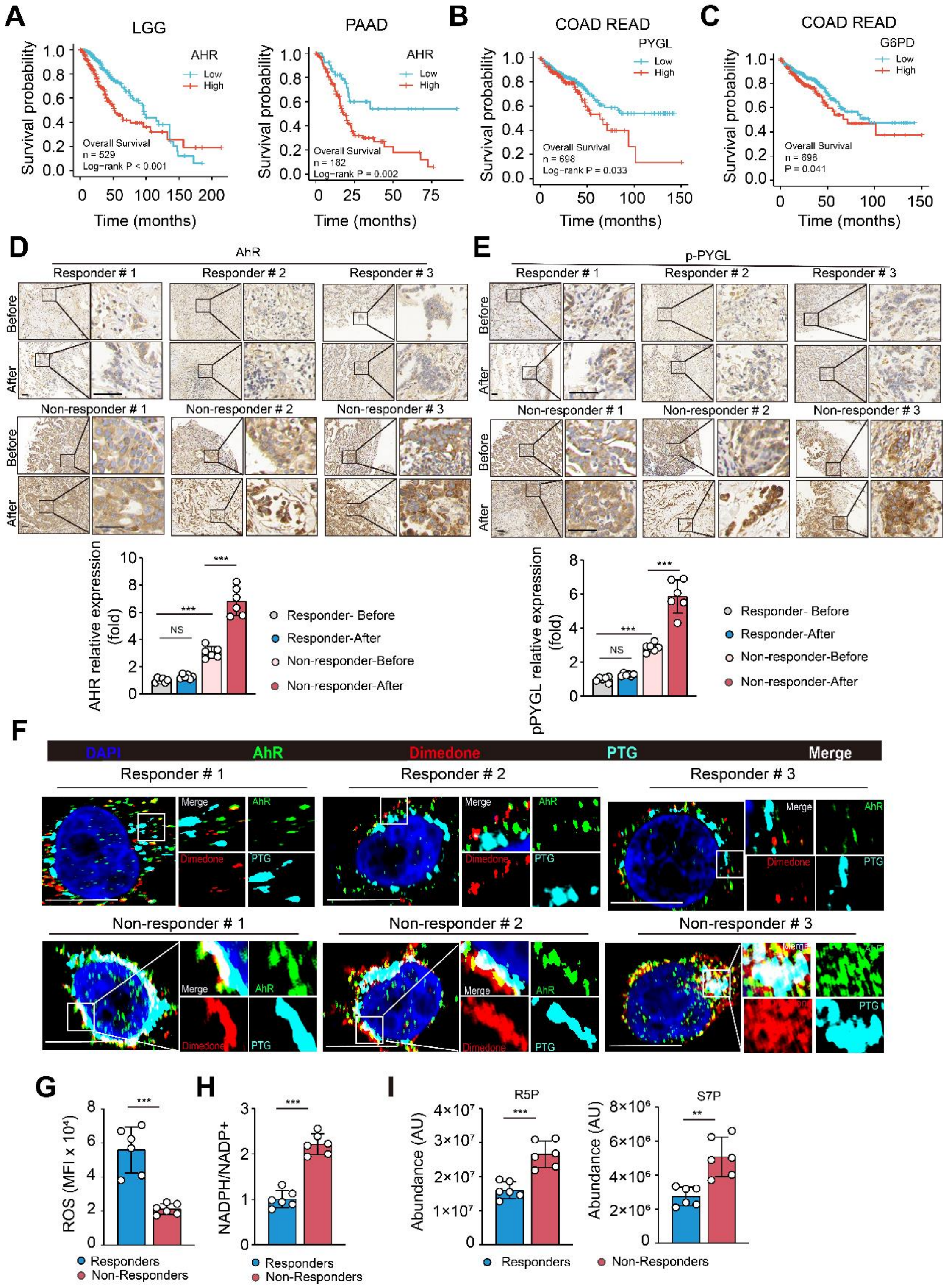

Fig. S8: AHR-glycogenolysis occurs in chemo-resistant cancer patients. Related to Fig. 7. (**A-C**) Overall survival compared to the AHR level in people with glioma (n = 529), and pancreatic adenocarcinoma (n = 182) (**A**), and PYGL level in people with colon cancer (n = 698) (**B**), and G6PD level in people with colon cancer (n = 698) (**C**). (**D** and **E**) The tissue sections from lung cancer patients including responders or non-

responders before and after chemotherapy were immunohistochemical staining with anti-AHR antibody (**D**) and anti-pPYGL antibody (**E**) Scale bars, 50 μm. (n = 6 per group). (**F**) Isolated primary tumor cells from fresh lung cancer tissues including responders and non-responders after chemotherapy were treated with dimedone (5 mM) for 30 min. The location of AHR (green), Dimedone (red) and STBD1 (cyan) in the isolated tumor cells of tumor tissues was observed under Super-Resolution Microscope. Scale bars, 10 μm. (n = 6 per group). (**G-I**) ROS levels (**G**), the ratio of NADPH/NADP+ (**H**) or R5P and S7P (**I**) in isolated primary tumor cells from fresh lung cancer tissues including responders and non-responders after chemotherapy were analyzed. (n = 6 per group). All error bars are mean ± SD, p values were calculated by one-way ANOVA followed by Bonferroni's test (**D** and **E**), two-tailed unpaired Student' s t test (**G-I**) and Log-rank test (**A-C**), $^{**}p < 0.01$, $^{***}p < 0.001$; NS, not significant ($p > 0.05$).

Supplementary Tables 1-4

Breast and lung cancer tissues were obtained by needle biopsy before chemotherapy, whereas after chemotherapy, tumor tissues were obtained by surgery. The chemotherapy responders and non-responders were evaluated according to the Response Evaluation Criteria in Solid Tumors (RECIST).

Table 1. Clinical information of breast cancer patients (paraffin section)

Patients NO.	Gender	Age	Status	Sample	Response Evalution
1	F	50	Newly diagnosis	Paraffin section	SD
2	F	55	Newly diagnosis	Paraffin section	SD
3	F	61	Newly diagnosis	Paraffin section	SD
4	F	62	Newly diagnosis	Paraffin section	SD
5	F	62	Newly diagnosis	Paraffin section	SD
6	F	49	Newly diagnosis	Paraffin section	SD
7	F	43	Newly diagnosis	Paraffin section	PR
8	F	67	Newly diagnosis	Paraffin section	PR
9	F	24	Newly diagnosis	Paraffin section	PR
10	F	61	Newly diagnosis	Paraffin section	PR
11	F	62	Newly diagnosis	Paraffin section	PR
12	F	53	Newly diagnosis	Paraffin section	PR

Table 2. Clinical information of lung cancer patients (parafin section)

Patients NO.	Gender	Age	Status	Sample	Response Evalution
1	F	70	Newly diagnosis	Paraffin section	SD
2	M	59	Newly diagnosis	Paraffin section	SD
3	F	43	Newly diagnosis	Paraffin section	SD
4	M	41	Newly diagnosis	Paraffin section	SD
5	M	68	Newly diagnosis	Paraffin section	SD
6	M	49	Newly diagnosis	Paraffin section	SD
7	F	59	Newly diagnosis	Paraffin section	PR
8	M	47	Newly diagnosis	Paraffin section	PR
9	M	66	Newly diagnosis	Paraffin section	PR
10	F	63	Newly diagnosis	Paraffin section	PR
11	F	41	Newly diagnosis	Paraffin section	PR
12	F	67	Newly diagnosis	Paraffin section	PR

Table 3. Clinical information of breast cancer patients (tumor tissue)

Patients NO.	Gender	Age	Status	Sample	Response Evalution
1	F	57	Newly diagnosis	Tumor tissue	SD
2	F	59	Newly diagnosis	Tumor tissue	SD
3	F	61	Newly diagnosis	Tumor tissue	SD
4	F	56	Newly diagnosis	Tumor tissue	SD
5	F	42	Newly diagnosis	Tumor tissue	SD
6	F	49	Newly diagnosis	Tumor tissue	SD
7	F	45	Newly diagnosis	Tumor tissue	PR
8	F	48	Newly diagnosis	Tumor tissue	PR
9	F	35	Newly diagnosis	Tumor tissue	PR
10	F	48	Newly diagnosis	Tumor tissue	PR
11	F	64	Newly diagnosis	Tumor tissue	PR
12	F	43	Newly diagnosis	Tumor tissue	PR

Table 4. Clinical information of lung cancer patients (tumor tissue)

Patients NO.	Gender	Age	Status	Sample	Response Evalution
1	F	35	Newly diagnosis	Tumor tissue	SD
2	F	56	Newly diagnosis	Tumor tissue	SD
3	F	64	Newly diagnosis	Tumor tissue	SD
4	M	57	Newly diagnosis	Tumor tissue	SD
5	M	69	Newly diagnosis	Tumor tissue	SD
6	F	65	Newly diagnosis	Tumor tissue	SD
7	F	44	Newly diagnosis	Tumor tissue	PR
8	M	70	Newly diagnosis	Tumor tissue	PR
9	F	69	Newly diagnosis	Tumor tissue	PR
10	M	64	Newly diagnosis	Tumor tissue	PR
11	F	47	Newly diagnosis	Tumor tissue	PR
12	F	49	Newly diagnosis	Tumor tissue	PR

Figure 1

Figure 1E

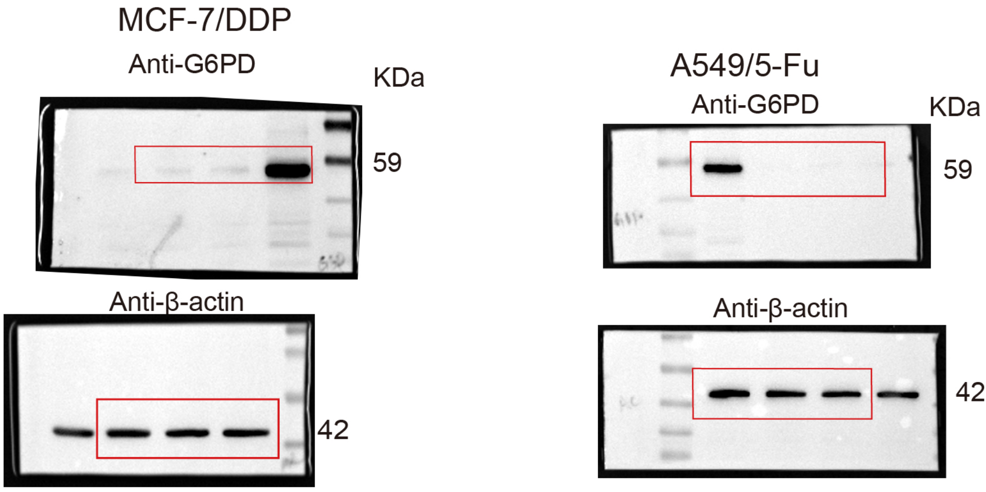

Figure S1F

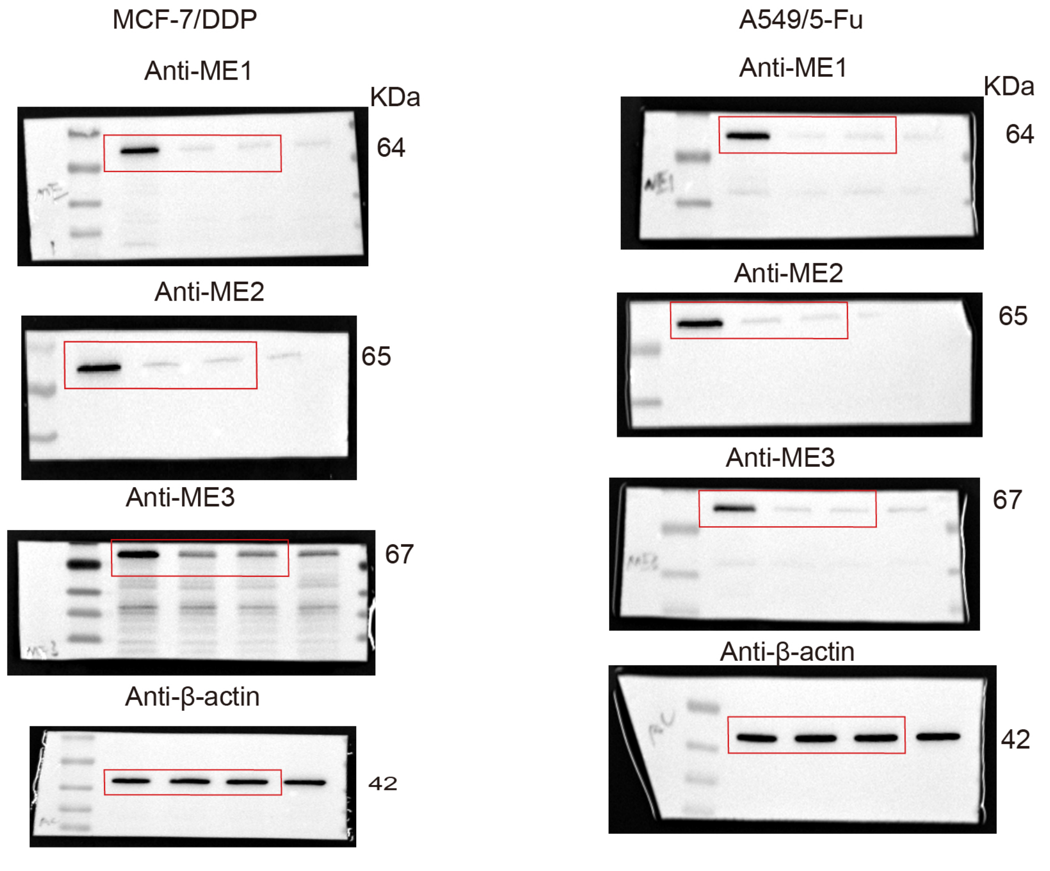

Figure S1H

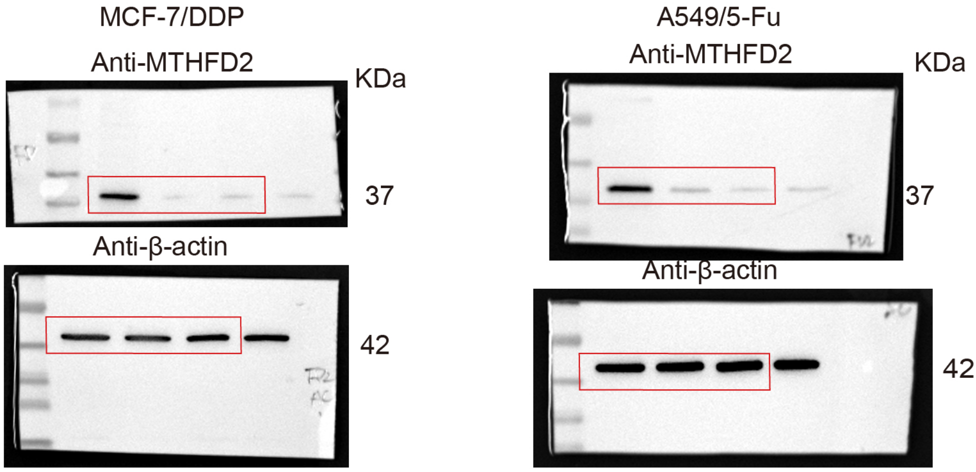

Figure 2

Figure 2F

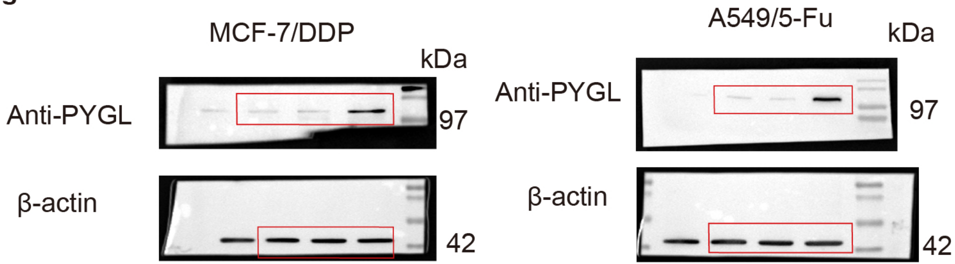

Figure S2D

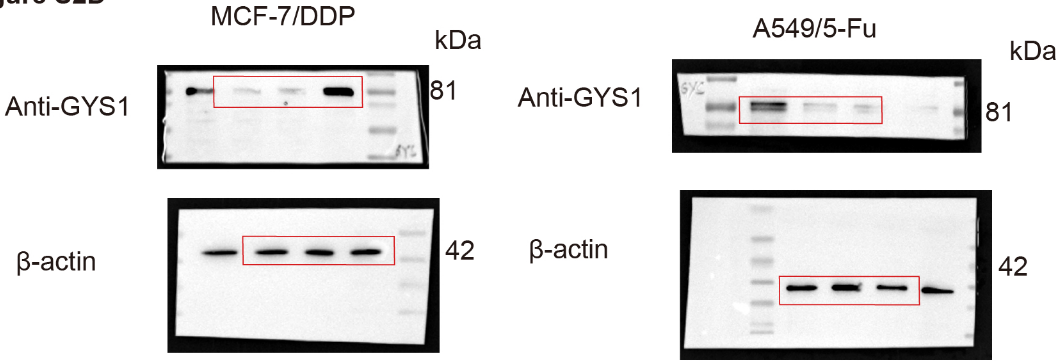

Figure 3

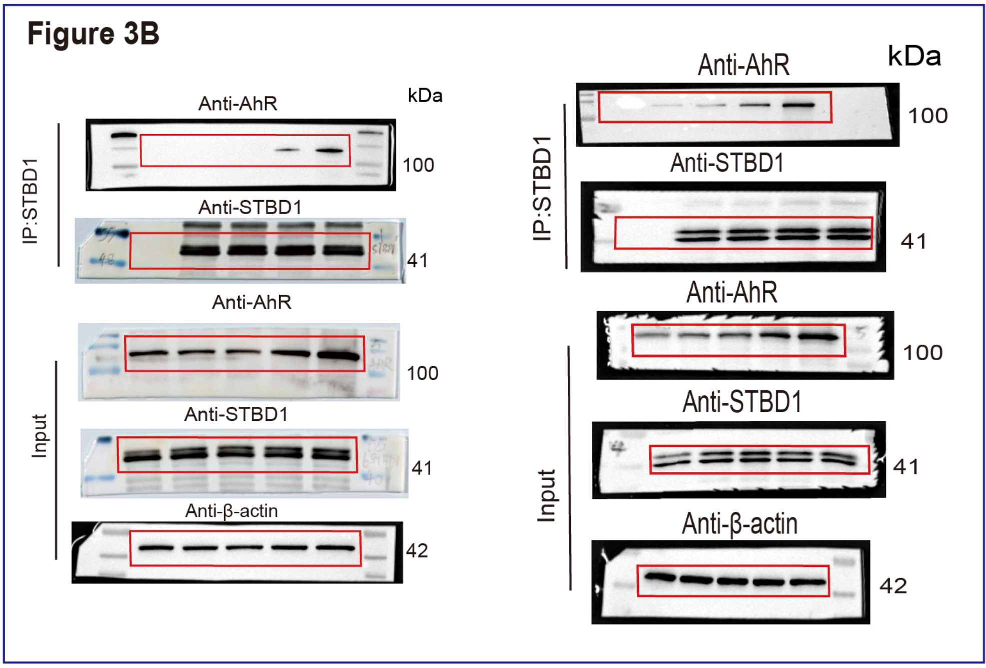
Figure 3B
Anti-AhR
kDa
IP:STBD1
100
Anti-STBD1
41
Anti-AhR
Input
100
Anti-STBD1
41
Anti-β-actin
42
Anti-AhR
kDa
IP:STBD1
100
Anti-STBD1
41
Anti-AhR
100
Anti-STBD1
Input
41
Anti-β-actin
42

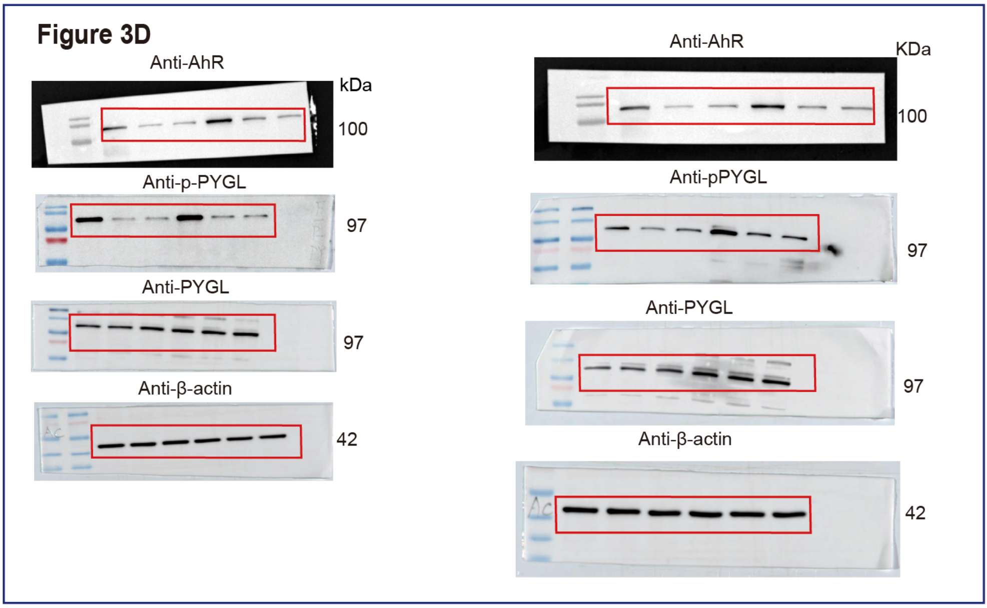
Figure 3D
Anti-AhR
kDa
100
Anti-p-PYGL
97
Anti-PYGL
97
Anti-β-actin
42
Anti-AhR
KDa
100
Anti-pPYGL
97
Anti-PYGL
97
Anti-β-actin
42

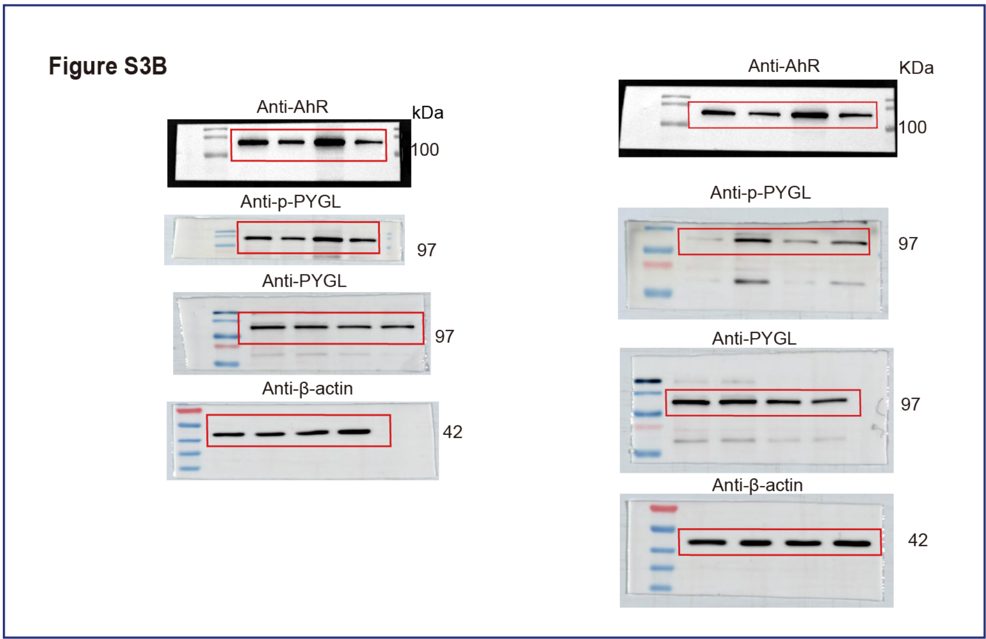
Figure S3B
Anti-AhR
kDa
100
Anti-p-PYGL
97
Anti-PYGL
97
Anti-β-actin
42
Anti-AhR
KDa
100
Anti-p-PYGL
97
Anti-PYGL
97
Anti-β-actin
42

Figure 4

Figure 4A

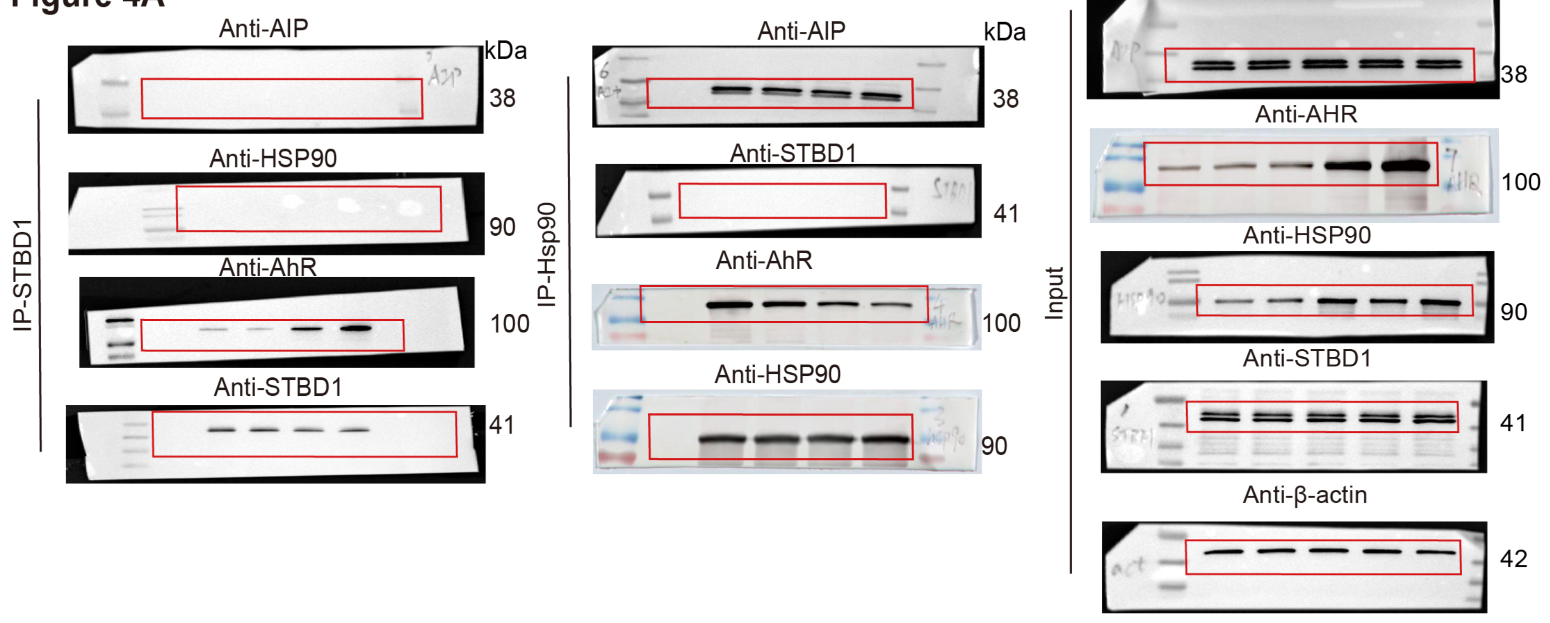

Figure 4D

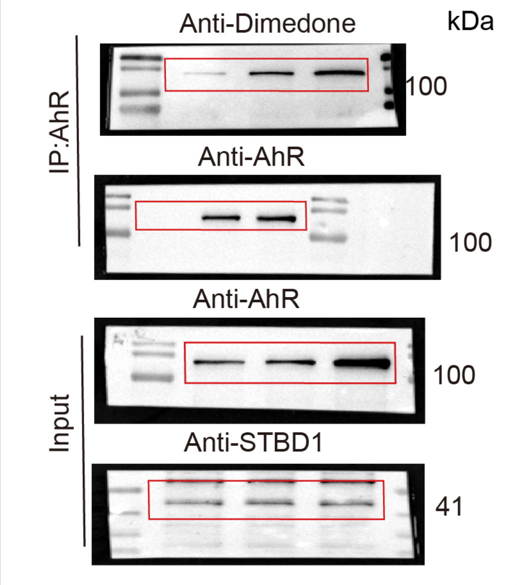

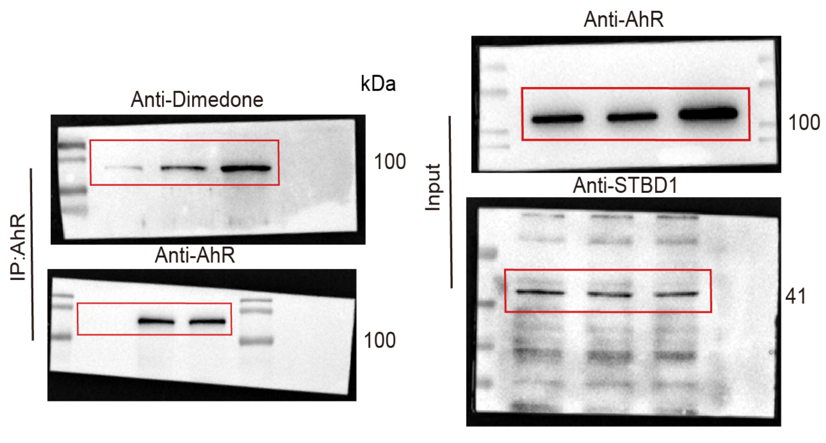

Figure 4E

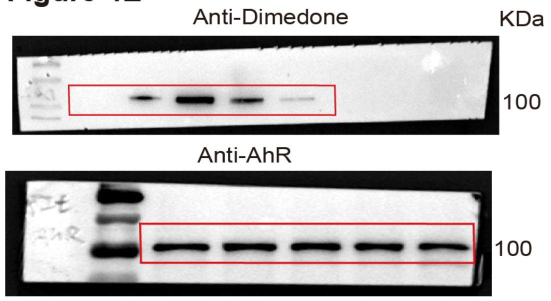

Figure 4G

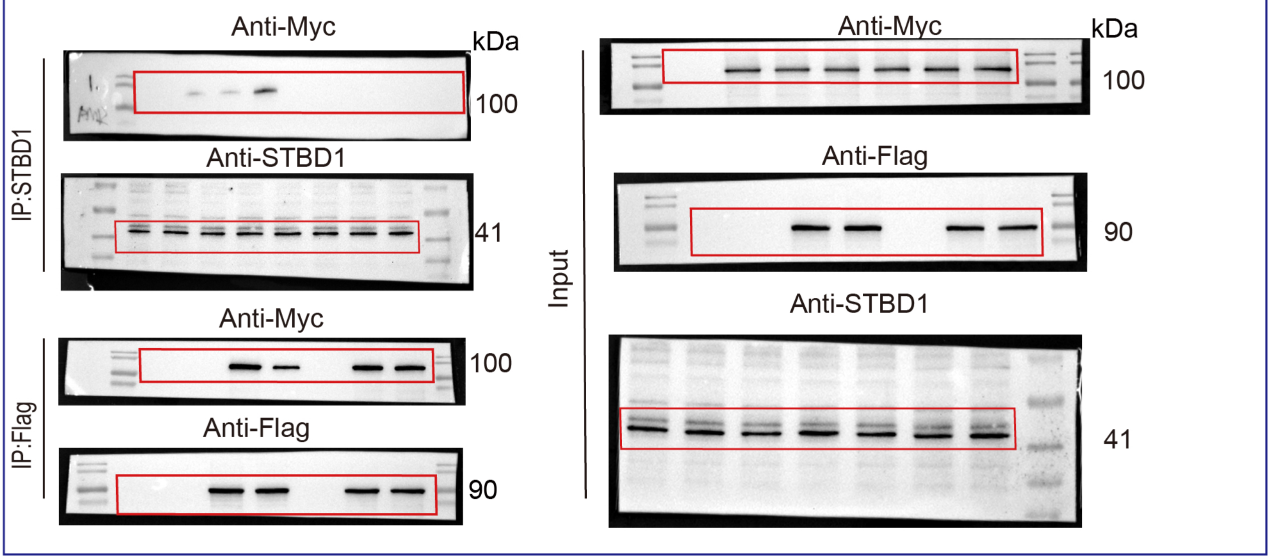

Figure S4

Figure S4A

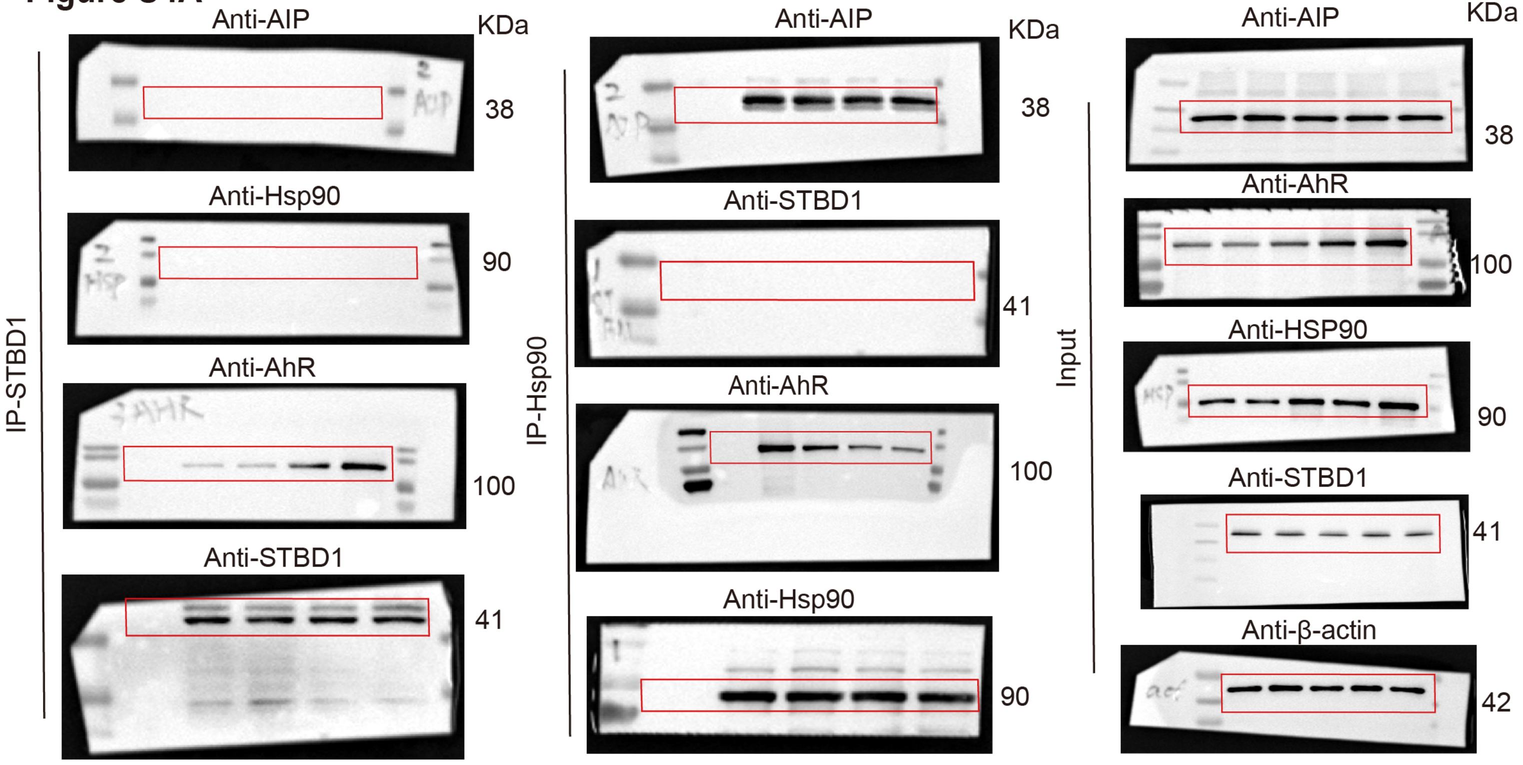

Figure S4C

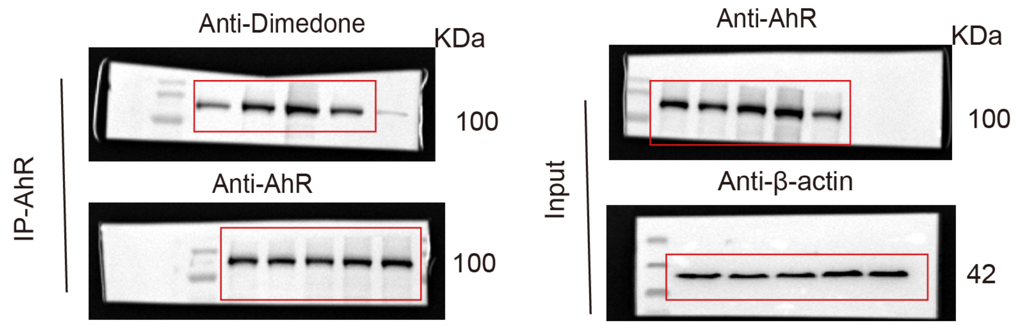

Figure 5

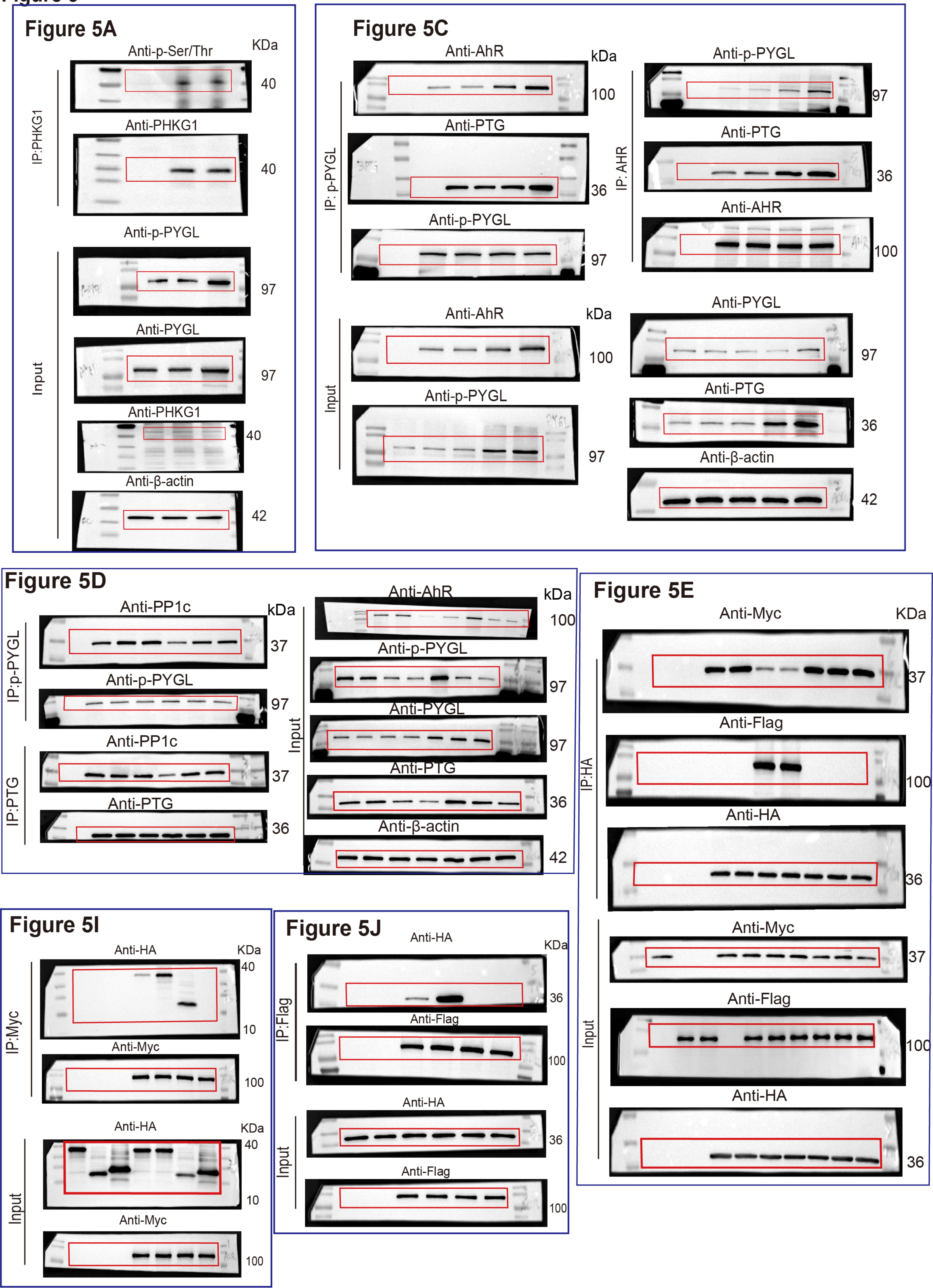

Figure 5A
Anti-p-Ser/Thr
KDa
IP:PHKG1
40
Anti-PHKG1
40
Anti-p-PYGL
Input
97
Anti-PYGL
97
Anti-PHKG1
40
Anti-β-actin
42
Figure 5C
Anti-AhR
kDa
IP: p-PYGL
100
Anti-PTG
36
Anti-p-PYGL
97
Anti-p-PYGL
IP: AHR
97
Anti-PTG
36
Anti-AHR
100
Anti-AhR
kDa
Input
100
Anti-p-PYGL
97
Anti-PYGL
97
Anti-PTG
36
Anti-β-actin
42
Figure 5D
Anti-PP1c
kDa
IP:p-PYGL
37
Anti-p-PYGL
97
Anti-PP1c
IP:PTG
37
Anti-PTG
36
Anti-AhR
kDa
100
Anti-p-PYGL
Input
97
Anti-PYGL
97
Anti-PTG
36
Anti-β-actin
42
Figure 5E
Anti-Myc
KDa
IP:HA
37
Anti-Flag
100
Anti-HA
36
Anti-Myc
Input
37
Anti-Flag
100
Anti-HA
36
Figure 5I
Anti-HA
KDa
IP:Myc
40
10
Anti-Myc
100
Anti-HA
KDa
Input
40
10
Anti-Myc
100
Figure 5J
Anti-HA
KDa
IP:Flag
36
Anti-Flag
100
Anti-HA
Input
36
Anti-Flag
100

Figure S5

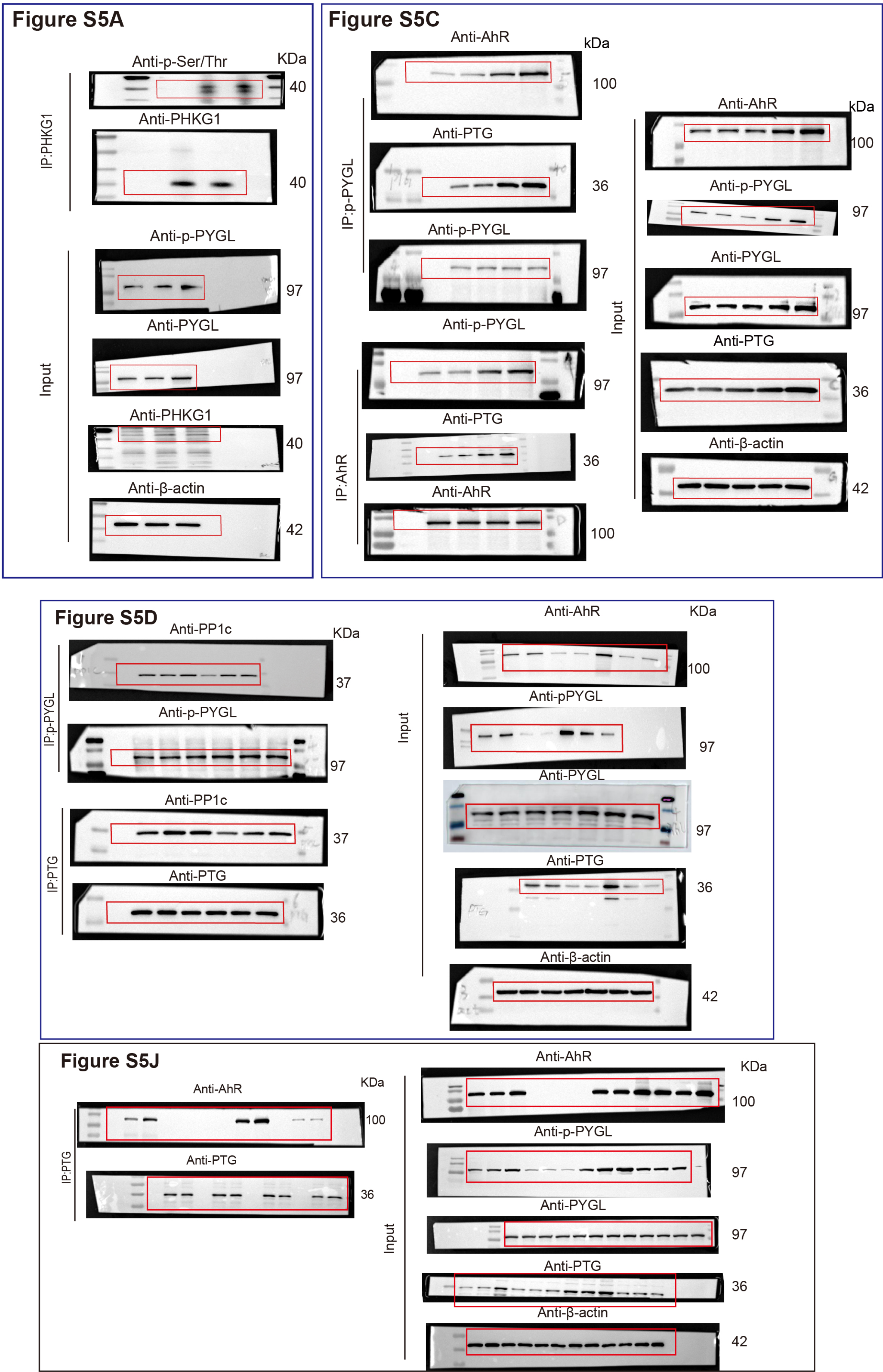
Figure S5A
Anti-p-Ser/Thr
KDa
40
Anti-PHKG1
40
IP:PHKG1
Anti-p-PYGL
97
Anti-PYGL
97
Anti-PHKG1
40
Anti-β-actin
42
Input
Figure S5C
Anti-AhR
kDa
100
Anti-PTG
36
Anti-p-PYGL
97
IP:p-PYGL
Anti-p-PYGL
97
Anti-PTG
36
Anti-AhR
100
IP:AhR
Anti-AhR
kDa
100
Anti-p-PYGL
97
Anti-PYGL
97
Anti-PTG
36
Anti-β-actin
42
Input
Figure S5D
Anti-PP1c
KDa
37
Anti-p-PYGL
97
IP:p-PYGL
Anti-PP1c
37
Anti-PTG
36
IP:PTG
Anti-AhR
KDa
100
Anti-pPYGL
97
Anti-PYGL
97
Anti-PTG
36
Anti-β-actin
42
Input
Figure S5J
Anti-AhR
KDa
100
Anti-PTG
36
IP:PTG
Anti-AhR
KDa
100
Anti-p-PYGL
97
Anti-PYGL
97
Anti-PTG
36
Anti-β-actin
42
Input

Figure S5-2

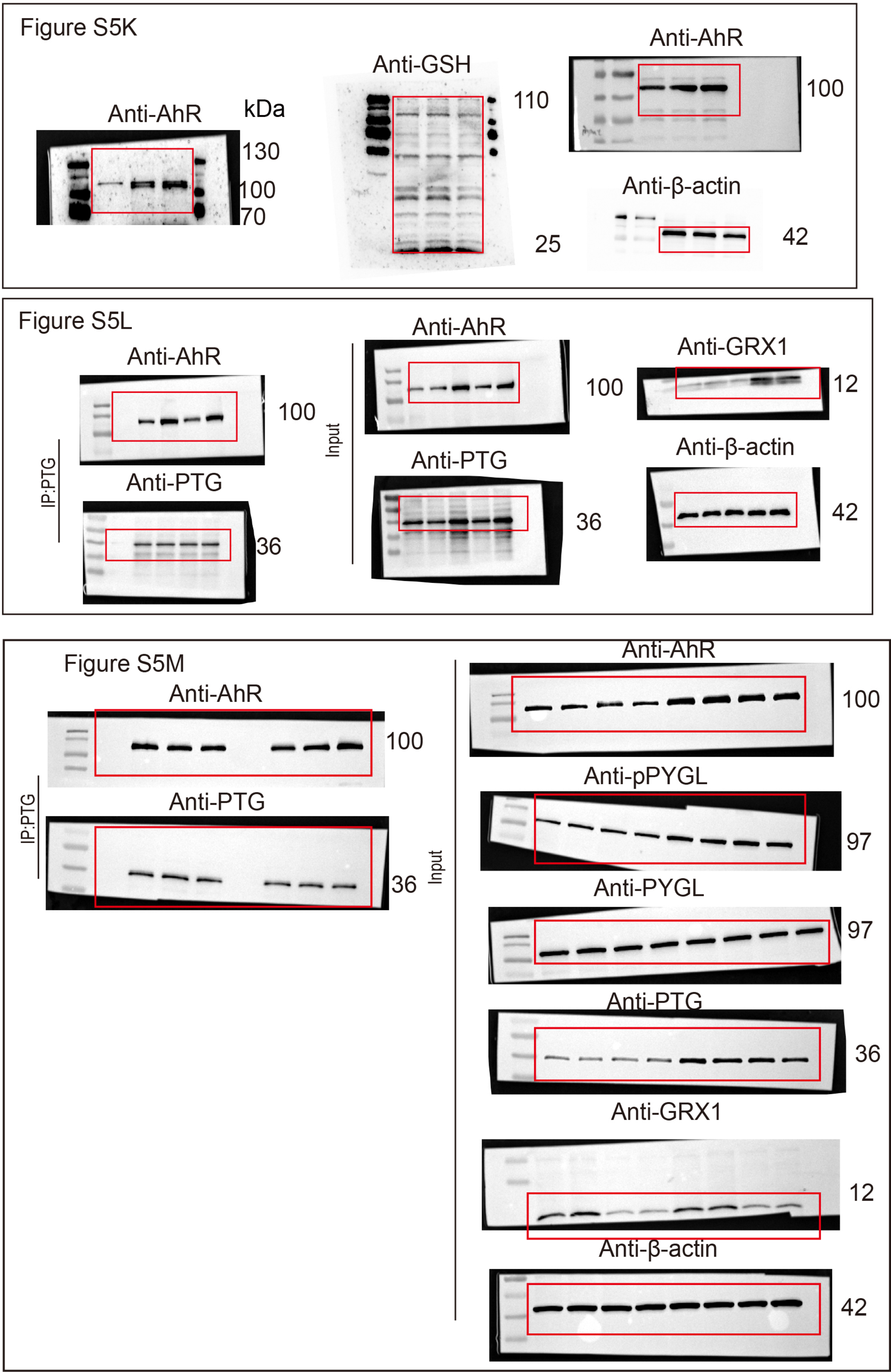

Figure S5K
Anti-AhR
kDa
130
100
70
Anti-GSH
110
25
Anti-AhR
100
Anti-β-actin
42
Figure S5L
Anti-AhR
100
Anti-PTG
36
IP:PTG
Input
Anti-AhR
100
Anti-PTG
36
Anti-GRX1
12
Anti-β-actin
42
Figure S5M
Anti-AhR
100
Anti-PTG
36
IP:PTG
Input
Anti-AhR
100
Anti-pPYGL
97
Anti-PYGL
97
Anti-PTG
36
Anti-GRX1
12
Anti-β-actin
42

Figure S7

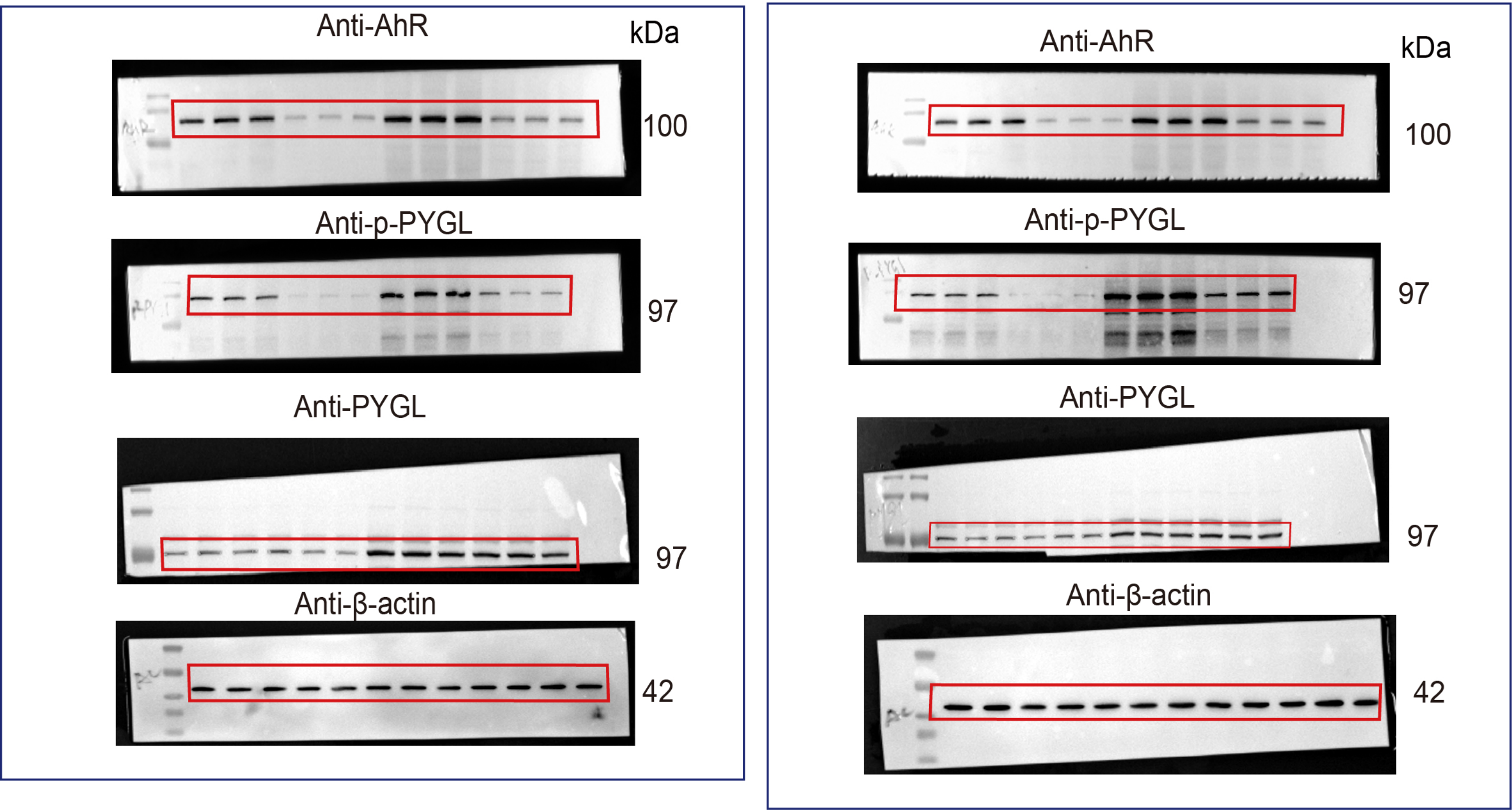

Anti-AhR
kDa
100
Anti-p-PYGL
97
Anti-PYGL
97
Anti-β-actin
42
Anti-AhR
kDa
100
Anti-p-PYGL
97
Anti-PYGL
97
Anti-β-actin
42
